# Valuing health-related quality of life using a hybrid approach: Tunisian value set for the EQ-5D-3L

**DOI:** 10.1007/s11136-020-02730-z

**Published:** 2021-01-14

**Authors:** Jaafar Chemli, Chema Drira, Hajer Felfel, Bram Roudijk, Fatima Al Sayah, Mokhtar Kouki, Amna Kooli, Myriam Razgallah Khrouf

**Affiliations:** 1 Faculty of Pharmacy of Monastir, University of Monastir, Monastir, Tunisia; 2 EuroQol Research Foundation, Rotterdam, Netherlands; 3 School of Public Health, University of Alberta, Edmonton, AB Canada; 4 High School of Statistics and Information Analysis, University of Carthage, Tunis, Tunisia; 5 Quality and Research, Tunis, Tunisia; 6 Pharmacy and Drug Department, Ministry of Health, Tunis, Tunisia

**Keywords:** EQ-5D-3L, Tunisian value set, Composite time trade-off, Discrete choice experiment, Health-related quality of life, Health measurement

## Abstract

**Objective:**

To develop a value set for EQ-5D-3L based on the societal preferences of the Tunisian population.

**Methods:**

A representative sample of the Tunisian general population was obtained through multistage quota sampling involving age, gender and region. Participants (*n* = 327), aged above 20 years, were interviewed using the EuroQol Portable Valuation Technology in face-to-face computer-assisted interviews. Participants completed 10 composite time trade-off (cTTO) and 10 discrete choice experiments (DCE) tasks. Utility values for the EQ-5D-3L health states were estimated using regression modeling. The cTTO and DCE data were analyzed using linear and conditional logistic regression modeling, respectively. Multiple hybrid models were computed to analyze the combined data and were compared on goodness of fit measured by the Akaike information criterion (AIC).

**Results:**

A total of 300 participants with complete data that met quality criteria were included. All regression models showed both logical consistency and significance with respect to the parameter estimates. A hybrid model accounting for heteroscedasticity presented the lowest value for the AIC among the hybrid models. Hence, it was used to construct the Tunisian EQ-5D-3L valuation set with a range of predicted values from − 0.796 to 1.0.

**Conclusion:**

This study provides utility values for EQ-5D-3L health states for the Tunisian population. This value set will be used in economic evaluations of health technologies and for Tunisian health policy decision-making.

**Supplementary Information:**

The online version of this article (10.1007/s11136-020-02730-z) contains supplementary material, which is available to authorized users.

## Background

Health Technology Assessment (HTA) has been increasingly used to inform healthcare policy decisions [1]. Through systematic evaluation of health interventions, decision-makers can support their policies in health care resource allocation by seeking evidence of efficiency in resource use. The World Health Organization recommends the use of HTA as a tool to support reimbursement decision-making and, e.g., in drug price-setting negotiations [2]. In addition, HTA can encourage a more efficient patient-centered approach when allocating resources by considering population-specific needs in decision-making [3].

The use of health economic evaluations in low- to middle-income countries remains limited due to a lack of expertise and to poor local data [4, 5]. In Tunisia, price negotiations for new therapies are part of the drug licensing process and involve many stakeholders such as the Ministry of Health, the Ministry of Trade, the National Health Insurance fund and the Tunisian central pharmacy. This process mainly uses external reference pricing as a benchmark. Since 1997, cost-effectiveness is one of the elements to be taken into consideration during drug licensing, price negotiations and reimbursement policy, especially for expensive and innovative drugs [6]. With the recent establishment of a national HTA agency, the National Authority for Assessment and Accreditation in Healthcare “INEAS”, Tunisia is taking a step towards implementing HTA in its health policy.

The quality-adjusted life year (QALY) is the standard outcome measure in health care economic evaluations, as recommended in current cost-utility analysis guidelines [7–9]. In this type of economic analysis, the incremental cost of a health technology is compared to the incremental health improvement expressed in QALYs, which can reflect the benefit of an intervention on both life expectancy and health-related quality of life (HRQOL). QALYs are calculated by multiplying life years by a correction factor: a health utility value attributed to each health state. The utility scale is anchored at 0 indicating a preference equal to immediate death, and 1 for preferences equal to full health. Negative values are assigned to states considered to be worse than dead (WTD). Utility weights are obtained by valuing how individuals perceive the quality of life associated with health states from a generic preference-based measure (GPBM). The most commonly used GPBM is EQ-5D [10]. This instrument was developed to measure, compare and value health status across disease areas [11]. The EQ-5D in its original version, EQ-5D-3L, defines (3^5^) = 243 different states in its descriptive system across five dimensions—mobility (MO), self-care (SC), usual activities (UA), pain or discomfort (PD), and anxiety or depression (AD)—each with three levels of problems (none, some and extreme problems/unable to) [11]. Every health state is described using a unique digit code, (e.g., ‘11122’ refers to a health state with no problems in MO, SC and UA, but some problems in PD and AD).

Utilities can be assigned to EQ-5D-3L health states by using a value set. These value sets or tariffs are constructed by eliciting preferences from the general population for the health states using a valuation method such as the Time Trade-Off (TTO) [12]. The EuroQol Group’s current protocol for the valuation of health states, the EQ-VT, uses composite Time Trade-Off (cTTO) and discrete choice experiments (DCE) to determine utility values for EQ-5D health states [13]. Although the EQ-5D-3L descriptive system has been translated into Arabic and validated during a previous study[14], its use in economic evaluation remains limited due to the absence of a value set in Tunisia and other Arabic-speaking countries. Acknowledging the logistical complexity of 5-Level valuation studies, we aimed to develop a value set for EQ-5D-3L based on the preferences of the Tunisian population as a first step towards patient-centered research.

## Methods

The study and the data collection were conducted in compliance with the EuroQol Group’s valuation protocol, EQ-VT [13]. Minor changes were made to the study design, as the protocol was originally designed for the EQ-5D-5L, and also in order to accommodate the local research teams’ constrained resources. As in previous EQ-5D valuation studies using the EQ-VT protocol, two preference elicitation techniques were used: cTTO and DCE. The questionnaire was administered via computer-assisted personal interviews using the EuroQol portable valuation technology v1.7 (EQ-PVT), power point-based software similar to the EQ-VT v 2.1 software [13]. Cyclic data quality control (QC) was performed to ensure interviewers’ compliance to the protocol [15]. The methods and analyses in this paper comply with the CREATE guidelines for reporting valuation studies of multiattribute utility‐based instruments [16].

### Sample selection and recruitment strategy

The EuroQol protocol requires a sample size of 300 participants [17]. In order to ensure an adequate number of valid responses, a larger sample of 350 respondents was targeted: non-institutionalized individuals from the general population, aged above 20 years and able to read, comprehend and complete the interviews, were eligible to participate in the study.

In order to preserve the representativeness of the population, multistage quota sampling was performed proportional to the region of residency. The national territory was divided into six regions following the Tunisian census: northwest, northeast, center east, center west, southeast and southwest. We also used a quota sampling in each region in terms of age and gender as both these factors showed evidence of being related to health state values [18, 19]. The sample quotas were based on the latest Tunisian population census of 2014 [20]. We used a mixed recruitment strategy, through the personal contacts of interviewers and their relatives and by direct approaches in public spaces such as coffee and other shops, and universities.

### Health state selection

Since the study combined cTTO and DCE tasks, an experimental hybrid design was developed in order to minimize the number of states and respondents required to obtain significant statistical estimates [13, 17]. The cTTO module included 28 health states, divided into 3 blocks of 10 EQ-5D-3L health states. Each block included the state ‘33333’ and 9 different states, at least one of them a mild state with only one deviation from full health. Eighteen of these health states were obtained using a 2-(3,5,2) orthogonal array distributed among the different blocks [21, 22]. Ten non-orthogonal states, including mild and intermediate states, were added according to block severity level balancing (i.e., the sum of health states severity index is equal in each block) and to minimum overlapping (i.e., reducing the number of health states with similar levels on the same attribute) [23].

The DCE module consisted of 60 pairs of states divided into 6 blocks. Health states were selected following the design developed by Stolk et al [24].

Each participant was randomly assigned to one of the DCE and TTO blocks. The order of the health states and choice pairs was randomized within the cTTO and DCE tasks. The feedback module was omitted to limit the interview time duration and reduce the risk of a participant dropping out of the interview.

### Eliciting preferences

Composite TTO uses two different approaches to value health states: conventional TTO for better than dead (BTD) health states and lead-time TTO (LT-TTO) for WTD states [25]. Following the QALY scale, health utility values range on a scale anchored at 0 (death) and 1 (full health) and bound to -1 for WTD states [26]. In conventional TTO, the utility value (u) ranges from 0 to 1, and is equal to ${{\text{x}} \mathord{\left/ {\vphantom {{\text{x}} {10}}} \right. \kern-\nulldelimiterspace} {10}}$ where x is the number of years in full health when the participant states the indifference point. For WTD health states, values are calculated taking into account the lead-time of 10 years, ${\text{u = }}{{\left( {{\text{x - 10}}} \right)} \mathord{\left/ {\vphantom {{\left( {{\text{x - 10}}} \right)} {10}}} \right. \kern-\nulldelimiterspace} {10}}$. Hence, u ranges from -1 (trading all years of the lead-time) to 0. During the cTTO task, an interactive visual scale aid allowed the participant to adjust to the number of years traded.

In the DCE task, participants were asked to choose their preferred alternative from two impaired EQ-5D-3L health states with no specification with respect to time duration.

Interviews started with a general presentation of the study and its purposes. Informed consent was obtained from all respondents and general background questions (i.e., age, sex and region of residency) were collected before starting the survey. Respondents were asked to report on their own health using the EQ-5D-3L questionnaire and the visual analogue scale (EQ VAS). Subsequently, they started the cTTO task and received instructions on how it works. The respondents first completed 3 wheelchair examples, in which both the BTD and the WTD tasks were shown to them. Next, the respondents completed three practice cTTO questions using EQ-5D-3L health states that were not included in the design. Finally, all respondents completed 10 cTTO tasks for health states from the block of health states they were assigned. Upon completion of the cTTO, the respondents received instructions on the DCE tasks, and subsequently completed 10 of these. Lastly, the respondents completed a socio-demographic questionnaire, after which they were thanked for their participation and debriefed. Each respondent received compensation.

### Quality control process

The quality control process enacted was based on the EuroQol QC protocol [15]. During the interviews, the EQ-PVT software allows the collection of metadata similar to that collected when using the EQ-VT software. Overall, the data were assessed for protocol compliance, interviewer effects, and for face validity.

**Fig. 1:**
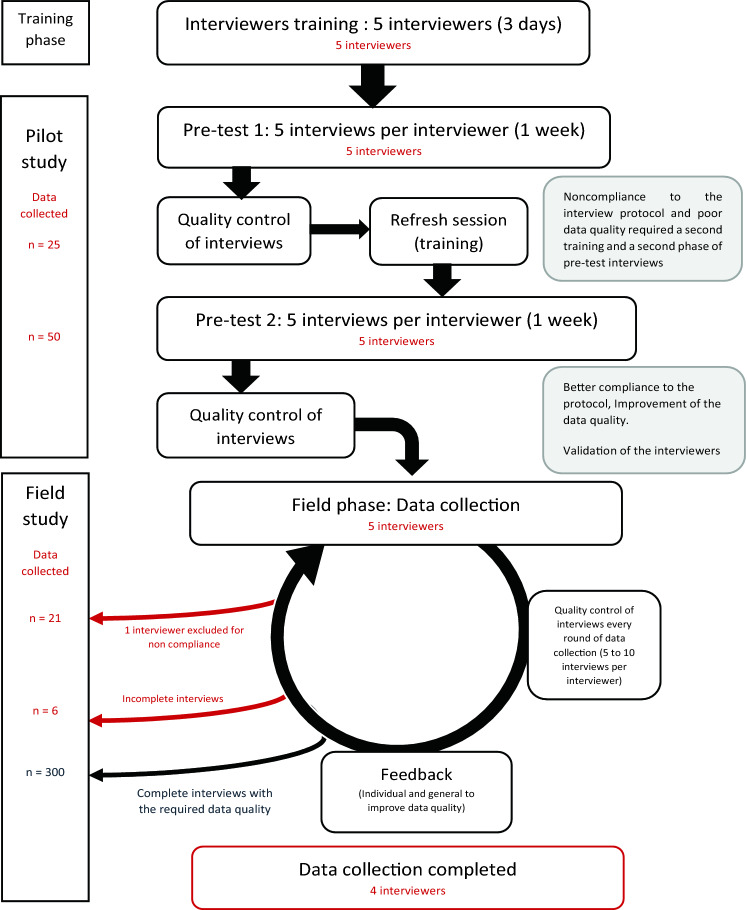
Data collection process and quality control of interviewers

Five interviewers were recruited among Pharm.D students at the Faculty of Pharmacy of Monastir. They were trained by the principal investigators (CD and HF) during a 3-day workshop using training materials provided by the EuroQol Group. After completing the training, each interviewer performed five practice interviews for which the data were retained if all the quality criteria in data collection were met, as shown in Fig. 1. After validation of the interviewers, continuous monitoring of data quality was performed through cyclic QC (i.e., QC after every round of data collection) by the EuroQol support team (BR and FAS). This was followed by a discussion including general and individual feedback of the interviewers’ performances.

### Statistical analysis

#### Sample characteristics

Descriptive statistics (frequencies and percentages) were used to report the sample socio-demographic characteristics (age group, region of habitat, gender, marital status, education level, employment status and health coverage) and compare them with national values. Self-reported health status on the EQ-5D-3L descriptive system and on the EQ VAS were also analyzed. To describe the participants’ responses to the preference elicitation tasks, we used the misery index score (MIS), defined as the sum of the attribute levels (e.g., for the health state ‘12321’ ${\text{MIS = 1 + 2 + 3 + 2 + 1 = 9}}$). This is a very crude measure, as there is no discrimination between the attributes, but it allowed us to compare health states by the number of deviations from full health. The responses to the cTTO tasks were analyzed: (i) by eliciting the value’s distribution for the overall responses, (ii) for the ‘pit’ state (‘33333’, MIS = 15), and (iii) by reporting the mean values observed per MIS. The DCE responses were described in terms of preference proportion by pair of health states and by MIS.

#### Data modeling

Regression models were estimated to determine values for all 243 health states described by EQ-5D-3L. The cTTO and DCE data were modeled separately first, and later combined into a hybrid regression model.

Multiple regression models for the cTTO data were estimated and compared in terms of prediction accuracy using the mean absolute error (MAE). The dependent variable was defined as disutility (i.e., 1 minus the cTTO utility value observed for a health state). Thus, coefficients expressed utility decrements of moving from level 1 (no problem) to upper levels. Disutility was explained by 10 independent variables associated with the coefficients representing the utility decrement of moving from level 1 to intermediate severity level (2) and extreme severity level (3) in each of the five EQ-5D dimensions. Dummy variables were named referring to the dimension and to the severity level associated with each (e.g., if the health state included extreme problems in usual activities UA2 would be equal to zero and UA3 to 1).

As the responses in the cTTO and DCE tasks were clustered within respondents, the cTTO data were analyzed using a random-intercept generalized least squares (GLS) model (Model 1). The mean and standard deviations of the observed cTTO values varied between the most severe health states and milder health states, which may have affected the data modeling. Hence, a homoscedasticity test was performed using the Breusch–Pagan Lagrange multiplier test [27]. As homoscedasticity was rejected, a model that corrected for multiplicative heteroscedasticity was estimated (Model 3) [13, 28]. In addition, a Tobit model censoring at − 1 (Model 2) was performed, since the cTTO task censors values below − 1 by limiting the possible time to trade to 10 years in the lead-time part of the task [29]^,^. Finally, a Tobit model censoring at -1 and accounting for heteroscedasticity was explored (Model 4). All model equations are reported in Appendix 1.

For the DCE observations, conditional logistic regression (Model A) was performed using the same cTTO model’s dummy parameters, and the dependent variable was the stated choice for each health state pair (i.e., 0 or 1 for the health state A of each pair, as the EQ-PVT randomly assigns the order of appearance of the pairs and their configuration). The model generated values on a latent arbitrary scale that required to be rescaled to produce QALYs, and was thus anchored on the utility range (0 death, 1 full health). We assumed that the DCE model coefficients were proportional to those of the cTTO model [17, 30]. Hence, a proportional rescaling parameter θ (theta)^1^ was introduced in order to allow comparison between the dichotomous and the continuous models [29].

Finally, hybrid models were estimated, using the same assumptions as in the cTTO models. Thus, a standard hybrid model (Model I), a hybrid Tobit model censoring at -1 (Model II), a hybrid model correcting for heteroscedasticity (model III), and a hybrid Tobit model correcting for multiplicative heteroscedasticity (Model IV) were estimated. The models were compared in terms of a set of criteria: logical consistency of the parameter estimates, significance of the parameters, and goodness of fit of the model measured by the Akaike information criterion (AIC).

Although each model estimated took the form of equation 1, details of the various models estimated are reported in the Appendix.

1 $$ {\text{Y = }}\beta {\text{0 + }}\beta {\text{1}} \times {\text{MO2 + }}\beta {\text{2}} \times {\text{MO3 + }}\beta {\text{3}} \times {\text{SC2 + }}\beta {\text{4}} \times {\text{SC3 + }}\beta {\text{5}} \times {\text{UA2 + }}\beta {\text{6}} \times {\text{UA3 + }}\beta {\text{7}} \times {\text{PD2 + }}\beta {\text{8}} \times {\text{PD3 + }}\beta {\text{9}} \times {\text{AD2 + }}\beta {\text{10}} \times {\text{AD3}}. $$

Statistical analyses were performed using STATA/MP 13.

## Results

### Data collection and quality check procedure

Data collection took place between June and September 2019. A total of 327 participants were interviewed nationwide. Interviewers were free not to include the data they collected if they felt that the participant clearly did not understand the task. One of the five interviewers was excluded for showing non-compliance with the interviewer’s protocol, leading to data quality issues. This did not improve over two consecutive data collection rounds. Subsequently, the data collected by this interviewer were excluded. Data monitoring indicated satisfactory data quality for the other interviewers. Six additional interviews were excluded for non-completion of the DCE or the cTTO tasks, resulting in 300 interviews to be included for data analysis.

### Sample characteristics

The study sample was generally representative of the Tunisian population in terms of age, gender and region of habitat, with a slight over-representation of the northeast region of the country and of male youths aged between 20 and 39 years old, as reported in Table 1.

**Table 1:** Characteristics of the respondents and their self-reported health status on EQ-5D-3L

Sampling characteristics	Study sample. n (%) (*n* = 300)	General population [19] (%)
**Sex**		
Male	154 (51.3)	49.8
Female	146 (48.7)	50.2
**Age group**		
20–29	86 (28.7)	25.3
30–39	74 (24.7)	23.2
40–49	53 (17.7)	19.1
50-59	46 (15.3)	15.7
60 +	41 (13.6)	16.8
**Region**		
Northeast	124 (41.3)	39.1
Northwest	36 (12.0)	10.6
West-central	33 (11.0)	12.8
East-central	64 (21.3)	23.1
Southwest	16 (5.4)	5.5
Southeast	27 (9.0)	8.9
**Civil state**		
Single	129 (43.0)	36.9
Married	134 (44.6)	56.6
Divorced	6 (2.0)	1.3
Widow	14 (4.7)	5.2
Missing data	17 (5.7)	
**Education**		
None	10 (3.4)	19
Primary school	26 (13)	32.8
High school	73 (21.0)	35.3
Higher education	172 (57.3)	12.9
Missing data	16 (5.3)	
**Activity**		
Unemployed	13 (4.2)	14.8
Active	197 (65.5)	46.5
Laborer	37 (12.3)	Not available
Part-time job	8 (2.7)	Not available
Employee	103 (34.3)	Not available
Executive officer	40 (13.2)	Not available
Liberal	9 (3.0)	Not available
Inactive	75 (25)	38.7
Housewife (house-husband)	13 (4.3)	Not available
Student	38 (12.7)	Not available
Retired	24 (8.0)	Not available
Missing data	15 (5.0)	
**Medical coverage**		
None	55 (18.3)	18.7
Yes	231 (77.0)	80.5
Other	0 (0)	0.8
Missing data	14 (4.7)	
Participants stating that their religious beliefs had an impact on the valuation task	43 (14.3)	Not applicable

*n* frequency, % percentage, *SD* standard deviation

No problems in any dimension of EQ-5D-3L (i.e., state ‘11111’) was self-reported by 83 participants (27.7%), 43 of whom (52.4%) gave a score of at least 90 on the EQ VAS, with a mean score equal to 83.91 (${\text{SD = 13}}{\text{.4}}$) and a median equal to 90. Overall, the mean observed EQ VAS score was equal to 72.7 and only 6% of the respondents reported a score lower than 50.

**Fig. 2:**
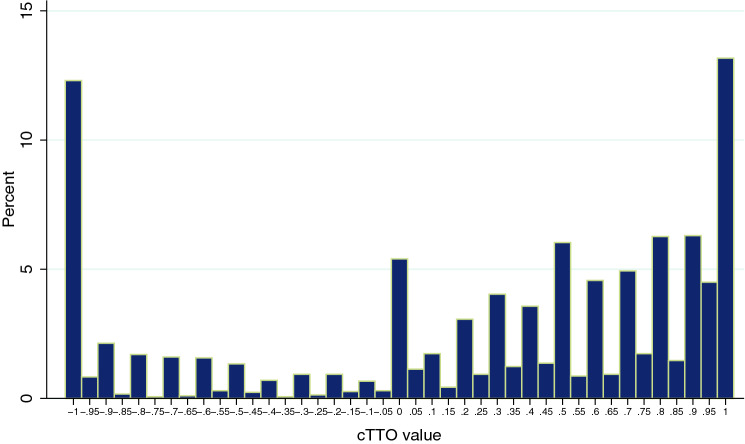
Distribution of the observed cTTO values in percentages

The distribution of the responses in the cTTO task is reported in Fig. 2, where 790 responses (26.3%) were negative and 369 (12.3%) of the observations equaled -1. In addition, cTTO values were clustered at the value 1, with 395 observations (13.2%). For 16 health states, observations ranged from -1 to 1, and from 0 to 1 for 5 other states. The mean cTTO value was negative for 8 health states with a lowest value for the state ‘33333’ of − 0.72.

For the DCE data, the observations show that participants chose the state with a lower MIS in 73.81% of their responses. For pairs of health states with an equal MIS, life A was chosen in 50.88% of the answers. The pair state number 21 showed a maximum in similar responses with 99.2% of respondents preferring life B.

### Modeling

All models derived logically consistent and significant parameter estimates (p < 0.05). For all cTTO models, the constant term was nearly zero and non-significant and was therefore suppressed. Model 1 presented the lowest AIC value; meanwhile Model 3 had the lowest MAE (see Table 2). Although Model 1 was the best-fitting model using AIC, it was not the most precise in terms of MAE.

**Table 2:** Parameter estimates and fit statistics of the cTTO models

	GLS (Model 1)	Tobit GLS (Model 2)	HET (Model 3)	Tobit HET (Model 4)
	β (SE)	β (SE)	β (SE)	β (SE)
MO2	0.086 (0.020)	0.074 (0.022)	0.055 (0.016)	0.088 (0.019)
MO3	0.546 (0.020)	0.592 (0.022)	0.565 (0.023)	0.548 (0.020)
SC2	0.161 (0.018)	0.168 (0.020)	0.142 (0.017)	0.164 (0.018)
SC3	0.398 (0.019)	0.439 (0.021)	0.396 (0.021)	0.397 (0.019)
UA2	0.103 (0.020)	0.094 (0.022)	0.070 (0.016)	0.103 (0.019)
UA3	0.246 (0.019)	0.274 (0.021)	0.262 (0.022)	0.247 (0.019)
PD2	0.054 (0.018)	0.047 (0.020)	0.052 (0.016)	0.054 (0.018)
PD3	0.299 (0.019)	0.322 (0.021)	0.308 (0.022)	0.300 (0.019)
AD2	0.094 (0.020)	0.088 (0.022)	0.086 (0.016)	0.092 (0.020)
AD3	0.280 (0.019)	0.300 (0.021)	0.283 (0.022)	0.277 (0.019)
Constant	0.000 (0.024)*	− 0.009 (0.027)*	0.018 (0.015)*	−0.007 (0.024)*
Uncensored Observations	3000	2631	3000	2631
Right-Censored Observations	0	369	0	369
AIC	3320.868	4193.709	3555.007	3308.948
BIC	3398.951	4271.792	3687.147	3447.095
MAE	0.341	0.342	0.335	0.342
Predicted value for the pit state	**− **0.769	**− **0.927	**− **0.814	−0.765

*cTTO* composite time trade-off, β coefficient , *SE* standard error , Dimensions : *MO* mobility , *SC* self-care , *UA* usual activities , *PD* pain/discomfort , *AD *anxiety/depression, *GLS* general least squares , *HET* model correcting for heteroscedasticity , *AIC* Akaike Information Criterion , *BIC* Bayesian Information Criterion, *MAE* Mean absolute error

*P value > 0.05

Coefficients estimated by the DCE model (Model A) were rescaled using the theta parameter. Model A showed the same ranking with respect to the dimensions, with a larger weight for the mobility dimension compared to the models for the cTTO data.

All the hybrid models (Table 3) showed both logical consistency and significance of the parameter estimates (p < 0.001 for all coefficients). Re-estimating the models with the constant term did not show any significant change in the parameter estimates as presented in the supplemental materials. Model III had the lowest AIC of the four hybrid models, equaling 6511.941. The difference with the hybrid Model I having the second lowest AIC value (ΔI =AIC_I_ – AIC_III_= 396.511), and calculating the likelihood of model III: ${\text{Exp}}\left( {{{{\text{ - 1}}} \mathord{\left/ {\vphantom {{{\text{ - 1}}} {2\Delta {\text{I}}}}} \right. \kern-\nulldelimiterspace} {2\Delta {\text{I}}}}} \right){\text{ = 7}}.{\text{92E - 87}}$ showed no evidence to support Model I and that there was no probability for the latter model to minimize the data information loss [31]. Furthermore, the same model showed the lowest MAE calculated on the basis of the cTTO observations. Hence, the hybrid model corrected for heteroscedasticity (Model III) was chosen to construct the Tunisian value set (supplemental material) using the following utility equation (2):

**Table 3:** Parameter estimates and fit statistics of the hybrid models

	HYBRID (Model I)	HYBRID TOBIT (Model II)	HYBRID HET (Model III)	HYBRID TOBIT HET (Model IV)
	β (SE)	β (SE)	β (SE)	β (SE)
MO2	0.082(0.014)	0.077(0.016)	0.076(0.012)	0.071(0.011)
MO3	0.587(0.015)	0.634(0.017)	0.597(0.016)	0.658(0.020)
SC2	0.177(0.013)	0.181(0.015)	0.165(0.012)	0.164(0.013)
SC3	0.338(0.014)	0.364(0.016)	0.340(0.015)	0.370(0.017)
UA2	0.082(0.015)	0.081(0.016)	0.078(0.012)	0.076(0.011)
UA3	0.244(0.014)	0.266(0.016)	0.251(0.014)	0.273(0.016)
PD2	0.052(0.014)	0.045(0.015)	0.057(0.012)	0.055(0.012)
PD3	0.270(0.014)	0.288(0.016)	0.276(0.014)	0.296(0.016)
AD2	0.095(0.015)	0.089(0.016)	0.095(0.012)	0.093(0.012)
AD3	0.329(0.014)	0.350(0.015)	0.332(0.014)	0.357(0.016)
AIC	6908.452	7788.361	6511.941	6937.185
BIC	6988.650	7868.560	6658.972	7084.215
MAE (cTTO observations as a benchmark)	0.342	0.343	0.341	0.344
Prediction for the pit state	− 0.768	− 0.902	− 0.796	− 0.954
Continuous uncensored observations	3000	2631	3000	2631
Continuous right-censored observations	0	369	0	369
Dichotomous observations	2903	2903	2903	2903

*cTTO* composite time trade-off, β coefficient, *SE* standard error, Dimensions: *MO* mobility, *SC* self-care, *UA* usual activities, *PD* pain/discomfort, *AD* anxiety/depression, *GLS* general least squares, *HET* model correcting for heteroscedasticity, *AIC* Akaike information criterion, *BIC* Bayesian information criterion, *MAE* mean absolute error

2 $$  {\text{U  =  1 - MO2}} \times {\text{0}}.{\text{076 - MO3}} \times {\text{0}}.{\text{597 - SC2}} \times {\text{0}}.{\text{165 - SC3}} \times {\text{0}}.{\text{34 - UA2}} \times {\text{0}}.{\text{078 - UA3}} \times {\text{0}}.{\text{251 - PD2}} \times {\text{0}}.{\text{057 - PD3}} \times {\text{0}}.{\text{276 - AD2}} \times {\text{0}}.{\text{095 - AD3}} \times {\text{0}}.{\text{332}}. $$

These coefficients express the utility decrement associated with each health problem, thus allowing the calculation of utilities for the 243 health states described in EQ-5D-3L. For example, the utility value associated with the health state ‘11223’ is equal to

3 $$ {\text{Utility}}({\text{11223}}){\text{ = 1 - }}\left( {{\text{0*0}}.{\text{076}}} \right){\text{ - }}\left( {{\text{0*0}}.{\text{597}}} \right) - \left( {{\text{0}} \times {\text{0}}.{\text{165}}} \right) - \left( {{\text{0}} \times {\text{0}}.{\text{34}}} \right) - \left( {{\text{1}} \times {\text{0}}.{\text{078}}} \right){\text{ - }}\left( {{\text{0}} \times {\text{0}}.{\text{251}}} \right) - \left( {{\text{1}} \times {\text{0}}.{\text{057}}} \right) - \left( {{\text{0}} \times {\text{0}}.{\text{276}}} \right) - \left( {{\text{0}} \times {\text{0}}.{\text{095}}} \right) - \left( {{\text{1}} \times {\text{0}}.{\text{332}}} \right){\text{ = 0}}.{\text{533}}. $$

## Discussion

In this study, a Tunisian value set for EQ-5D-3L was developed using a hybrid approach in accordance with EuroQol Group recommendations, thus (i) facilitating its utilization in international cross-country studies, and (ii) allowing comparisons between Tunisian valuations and those of other countries.

**Fig. 3:**
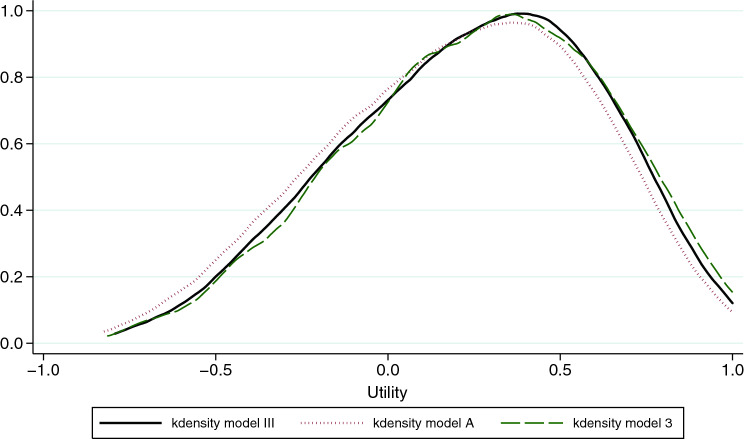
The kernel density plots of the prediction value for the 243 health states using the single models and the hybrid model. Model III: Hybrid model corrected for heteroscedasticity; Model A: Conditional logistic model. Model 3: Generalized Least Square model corrected for heteroscedasticity

The hybrid approach assumes that a single utility function underlies a participant’s responses, and estimating optimal parameters for the combined data would require the creation of a single likelihood function by combining the likelihood functions of the continuous and dichotomous responses (i.e., cTTO and DCE data) [20, 29]. Hence, we assumed the best-fitting continuous model could produce the best-fitting hybrid model, so we compared the cTTO models first, then the hybrid models. The assumption was refuted as model III was the best-fitting hybrid model (while Model 1 was the best-fitting cTTO model). The kernel density plots of the prediction value for the 243 health states, using the different single models (3 and A), showed high convergence between the cTTO and the DCE predictions, as shown in Fig. 3. This reflects the complementarity of using both elicitation techniques in order to produce estimates that were more consistent and to reach a closer utility function to the real one. The utility range varied from [− 0.814 to 1] for Model 3, [− 0.821 to 1] for Model A and [− 0.796 to 1] for Model III (see Table 4). Combining the data produced a narrower range resulting in a closer prediction of the pit state to the mean of the observed values which equaled − 0.727.

**Table 4:** Parameter estimates of the single models and hybrid model used to construct the value set

	HET GLS (Model 3)	CLOGIT (Model A)		Hybrid HET (Model III)
	β (SE)	β (SE)	Rescaled β	β (SE)
MO2	0.055(0.016)	0.397(0.078)	0.109	0.076(0.012)
MO3	0.565(0.023)	2.279(0.103)	0.629	0.597(0.016)
SC2	0.142(0.017)	0.676(0.074)	0.186	0.165(0.012)
SC3	0.396(0.021)	1.151(0.091)	0.318	0.340(0.015)
UA2	0.070(0.016)	0.341(0.082)	0.094	0.078(0.012)
UA3	0.262(0.022)	0.863(0.083)	0.238	0.251(0.014)
PD2	0.052(0.016)	0.220(0.072)	0.060	0.057(0.012)
PD3	0.308(0.022)	0.942(0.085)	0.260	0.276(0.014)
AD2	0.086(0.016)	0.386(0.086)	0.106	0.095(0.012)
AD3	0.283(0.022)	1.364(0.094)	0.376	0.332(0.014)
Constant	0.018(0.015)*			
Continuous observations	3000	0		3000
Dichotomous observations	0	2903		2903
Estimated values by health state				
U(11121)	0.948	0.94		0.943
U(32132)	− 0.101	− 0.181		− 0.133
U(33333)	− 0.814	− 0.821		− 0.796

*cTTO* composite Time Trade-off , *DCE* discrete choice experiments , β coefficient , *SE* standard error , Dimensions : *MO* mobility , *SC* self-care , *UA* usual activities , *PD* pain/discomfort , *AD* anxiety/depression , *GLS* general least squares , *HET* model correcting for heteroscedasticity

^*^P value > 0.05

In all models, MO3 had the highest weight (0.597), followed by SC3 (0.340), suggesting that mobility is by far the most important health dimension based on Tunisian preferences. This reflects how the public perceive physical disability and could be explained by the social disparities facing disabled people in Tunisia (i.e., discrimination in employment, lower access to education, and reduced quality of life) [32, 33]. The predicted value for the pit state (− 0.796) was lower than the ones observed in the value sets for high-income countries such as the USA or France (− 0.573 and − 0.525), but was similar to other low- to middle-income countries such as Indonesia or Ethiopia (− 0.865 and − 0.718), possibly reflecting divergences in health perceptions between countries with different income levels [34–37].

Modeling in our study did not account for religious beliefs, as suggested by some authors who considered participants’ answers in Christian and Muslim communities to be biased by their beliefs [38, 39]. Although 43 participants out of the 300 stated that their religious beliefs affected their responses, only one non-trader was reported during the valuation study. Furthermore, the Tunisian value set had lower utility values when compared with Iran’s value set which was presumed to be influenced by religion [40]. It may be necessary to explore whether religion should be accounted for in culturally religious communities during valuation studies.

Applying the existing crosswalk methodology developed by Van Hout *et al* to the Tunisian EQ-5D-3L value set allowed values to be estimated for the 3125 EQ-5D-5L health states and these are presented in the supplemental materials [41]. We observed similarities with recent international crosswalk studies, notably those of Sri Lanka and Poland [42, 43]. The Tunisian crosswalk value set has proportionally fewer health state utilities lower than zero or higher than 0.8 when compared with EQ-5D-3L tariff values. It has been suggested that this effect was due to restricting the range of index values in order to obtain an equivalent severity scope [43].

The main limitation of our study was that the sample may not have been fully representative of the population, as illiterate participants were not included the study. Illiterate people represent 18.8% of the general population in Tunisia (22.34% for those over 20 years), but since the ability to read is required to complete the valuation tasks, it was impossible to include them in the sample [20]. Another limitation is that, although our recruitment strategy covered all the nation’s regions, the quota sampling did not account properly for rural areas. Finally, due to technical issues with EQ-PVT, 97 DCE observations were corrupted and the data concerned were lost, which corresponded to a loss of DCE data of roughly 10 interviews.

## Conclusion

This is the first Tunisian preference-based value set to be published. These EQ-5D-3L health state utilities reveal the preferences of a sample of the Tunisian general population with respect to different impaired health states. The effect of having impaired mobility on HRQoL was the largest of all 5 dimensions. The Tunisian EQ-5D-3L values differed from those derived in other countries. This EQ-5D-3L value set should be considered for utilization in HTA in order to assist health policy decision-making in Tunisia.

## Supplementary Information

Below is the link to the electronic supplementary material.

(DOCX 20 kb)

(DOCX 21 kb)

(DOCX 79 kb)

(DOCX 14 kb)

## Data Availability

The datasets generated during and/or analyzed during the current study are available from the corresponding author on reasonable request.

## Footnotes

1 The logit coefficients of the dichotomous model β′ are not on the same scale as the coefficients of the continuous model β. As such the rescaling parameter θ is calculated in the hybrid modeling, and the estimated relationship between both coefficients is equal to β' = β /θ. This theta parameter was estimated in the hybrid models discussed in the methods section, which was used to rescale the DCE coefficients in the hybrid models.

## References

[1] International Network of Agencies for Health Technology Assessment. The INAHTA Working Group on HTA Impact. Published evidence on the influence of health technology assessment - A systematic review. Alberta: INAHTA; 2014.

[2] World Health Organization. Guideline on country pharmaceutical pricing policies [Internet]. Geneva: World Health Organization; 2013 [cited 2019 Sep 20]. http://www.ncbi.nlm.nih.gov/books/NBK258631/

[3] World Health Organization (WHO). More health for the money. In: WHO. Health systems financing: the path to universal coverage. [Internet]. 2010 [cited 2020 Feb 17]. Available from: https://www.who.int/whr/2010/10_chap04_en.pdf?ua=1.

[4] Singer ME (2009). Developing nations special issue. PharmacoEconomics.

[5] Babar ZUD, Scahill S (2010). Is there a role for pharmacoeconomics in developing countries?. PharmacoEconomics.

[6] République Tunisienne. Arrêté du ministre de la santé publique du 9 juin 1987, fixant la composition et le fonctionnement du comité technique des spécialités pharmaceutiques, en vue de l'autorisation de mise sur le marché, tel que modifié par l’arrêté du 6 juin 1990. Journal Officiel du 15 Juin 1990.

[7] Haute Autorité de Santé (HAS)**.** Choix méthodologiques pour l’évaluation économique à la HAS. Paris, HAS; 2011.

[8] European Network for Health Technology Assessment. Methods for health economic evaluations - A guideline based on current practices in Europe. 2015. https://eunethta.eu/wp-content/uploads/2018/03/Methods_for_health_economic_evaluations.pdf. Accessed 19 Nov 2019.

[9] National Institute for Health and Care Excellence (NICE). Single technology appraisal: user guide for company evidence submission template. [Internet]. 2015. https://www.nice.org.uk/process/pmg24/chapter/cost-effectiveness. Accessed 20 Mar 2020.

[10] Brazier JE, Rowen D, Lloyd A, Karimi M (2019). Future directions in valuing benefits for estimating QALYs: Is time up for the EQ-5D?. Value Health.

[11] Devlin NJ, Brooks R (2017). EQ-5D and the EuroQol Group: Past, present and future. Applied Health Economics and Health Policy.

[12] Torrance GW (1987). Utility approach to measuring health-related quality of life. Journal of Chronic Diseases.

[13] Stolk E, Ludwig K, Rand K, van Hout B, Ramos-Goñi JM (2019). Overview, update, and lessons learned from the international EQ-5D-5L valuation work: Version 2 of the EQ-5D-5L valuation protocol. Value Health.

[14] Aburuz S, Bulatova N, Twalbeh M, Gazawi M (2009). The validity and reliability of the Arabic version of the EQ-5D: a study from Jordan. Annals of Saudi Medicine.

[15] Ramos-Goñi JM, Oppe M, Slaap B, Busschbach JJV, Stolk E (2017). Quality control process for EQ-5D-5L valuation studies. Value Health.

[16] Xie F, Pickard AS, Krabbe PFM, Revicki D, Viney R, Devlin N (2015). A checklist for reporting valuation studies of multi-attribute utility-based instruments (CREATE). PharmacoEconomics.

[17] Oppe, M., & Van Hout, B. (2017). The “power” of eliciting EQ-5D-5L values: the experimental design of the EQ-VT. EuroQol Working Paper Series. Rotterdam: EuroQol Research Foundation.

[18] Hausman DM (2006). Valuing Health. Philosophy & Public Affairs.

[19] Wittenberg E, Halpern E, Divi N, Prosser LA, Araki SS, Weeks JC (2006). The effect of age, race and gender on preference scores for hypothetical health states. Quality of Life Research.

[20] Institut national de la statistique. Résultats recensement Tunisie 2014 [Internet]. 2014. http://www.ins.tn/fr/resultats

[21] Yang Z, Luo N, Bonsel G, Busschbach J, Stolk E (2018). Selecting health states for EQ-5D-3L valuation studies: Statistical considerations matter. Value Health..

[22] Yang Z, Luo N, van Busschbach J, Stolk E (2016). Using orthogonal design in selecting health states for the construction of EQ-5D-3L value set. Value Health..

[23] Law EH, Pickard AS, Xie F, Walton SM, Lee TA, Schwartz A (2018). Parallel valuation: A direct comparison of EQ-5D-3L and EQ-5D-5L societal value sets. Med Decis Mak..

[24] Stolk EA, Oppe M, Scalone L, Krabbe PFM (2010). Discrete choice modeling for the quantification of health states: The case of the EQ-5D. Value Health.

[25] Oppe M, Rand-Hendriksen K, Shah K, Ramos-Goñi JM, Luo N (2016). EuroQol protocols for time trade-off valuation of health outcomes. PharmacoEconomics.

[26] Patrick DL, Starks HE, Cain KC, Uhlmann RF, Pearlman RA (1994). Measuring preferences for health states worse than death. Medical Decision Making.

[27] Cameron AC, Trivedi PK (2010). Microeconometrics using stata.

[28] Feng Y, Devlin NJ, Shah KK, Mulhern B, van Hout B (2018). New methods for modelling EQ-5D-5L value sets: An application to English data. Health Economics.

[29] Ramos-Goñi JM, Craig B, Oppe M, van Hout B. Combining continuous and dichotomous responses in a hybrid model. EuroQol working paper series. [Internet] 2016. https://euroqol.org/wp-content/uploads/working_paper_series/EuroQol_Working_Paper_Series_Manuscript_16002_-_Juan_Ramos-Goni.pdf.

[30] Ramos-Goñi J, Pinto-Prades J, Oppe M, Cabasés J, Serrano-Aguilar P, Rivero-Arias O (2017). Valuation and modeling of EQ-5D-5L health states using a hybrid approach. Medical Care..

[31] Burnham KP, Anderson DR (2002). Model selection and multimodel inference: A practical information-theoretic approach.

[32] Trani JF, Bakhshi P, Lopez D, Gall F, Brown D (2017). La situation socioéconomique des personnes en situation de handicap au Maroc et en Tunisie : inégalités, coût et stigmatisation. Alter..

[33] Trani JF, Bakhshi P, Tlapek SM, Lopez D, Gall F (2015). Disability and poverty in morocco and tunisia: A multidimensional approach. Journal of Human Development and Capabilities.

[34] Pickard AS, Law EH, Jiang R, Pullenayegum E, Shaw JW, Xie F (2019). United states valuation of EQ-5D-5l health states using an international protocol. Value Health.

[35] Andrade LF, Ludwig K, Goni JMR, Oppe M, de Pouvourville G (2020). A french value set for the EQ-5D-5L. PharmacoEconomics..

[36] Purba FD, Hunfeld JAM, Iskandarsyah A, Fitriana TS, Sadarjoen SS, Ramos-Goñi JM (2017). The indonesian EQ-5D-5L value set. Pharmacoeconomics.

[37] Welie AG, Gebretekle GB, Stolk E, Mukuria C, Krahn MD, Enquoselassie F (2019). Valuing health state: An EQ-5D-5L value set for ethiopians. Value Health Regional Issues..

[38] Elbarazi I, Devlin NJ, Katsaiti M-S, Papadimitropoulos EA, Shah KK, Blair I (2017). The effect of religion on the perception of health states among adults in the United Arab Emirates: a qualitative study. BMJ Open..

[39] Jakubczyk M, Golicki D, Niewada M (2016). The impact of a belief in life after death on health-state preferences: True difference or artifact?. Quality of Life Research.

[40] Goudarzi R, Sari AA, Zeraati H, Rashidian A, Mohammad K, Amini S (2019). Valuation of quality weights for EuroQol 5-Dimensional health states with the time trade-off method in the capital of Iran. Value Health Regional Issues.

[41] Van Hout B, Janssen MF, Feng YS, Kohlmann T, Busschbach J, Golicki D (2012). Interim scoring for the EQ-5D-5L: Mapping the EQ-5D-5L to EQ-5D-3L value sets. Value Health.

[42] Golicki D, Nieewada M, Van Hout B, Janssen MF, Pickard SA (2014). Interim EQ-5D-5L value set for Poland: First crosswalk value set in central and eastern Europe. Value Health Regional Issues.

[43] Sanjeewa K, Gang C, Byrmesh J, Scuffham PA (2017). Mapping Sri Lankan EQ-5D-3L to EQ-5D-5L value sets. Value Health Regional Issues.

