# Supplementary material for: Valuing health-related quality of life using a hybrid approach: Tunisian value set for the EQ-5D-3L
Source: Qual Life Res. 2021 Jan 14;30(5):1445–55. doi: 10.1007/s11136-020-02730-z (PMC8068700; doi:10.1007/s11136-020-02730-z)
Supplement: Supplementary file 1 — (DOCX 20 kb) [file 11136_2020_2730_MOESM1_ESM.docx]

Parameter estimates for the hybrid model corrected for heterscedasticity modeled with the constant term

|  | Coef | Std. Err. | z | P>z | [95% Conf. Interval] | |
| --- | --- | --- | --- | --- | --- | --- |
| _mo2 | 0,0762727 | .0122551 | 6.22 | 0.000 | .052253 | .1002923 |
| _mo3 | 0,5979824 | .0163685 | 36.53 | 0.000 | .5659008 | .6300641 |
| _sc2 | 0,1655756 | .0123947 | 13.36 | 0.000 | .1412824 | .1898687 |
| _sc3 | 0,3409664 | .0148092 | 23.02 | 0.000 | .3119409 | .3699918 |
| _ua2 | 0,0788198 | .0122429 | 6.44 | 0.000 | .0548242 | .1028154 |
| _ua3 | 0,2510832 | .0144146 | 17.42 | 0.000 | .2228311 | .2793353 |
| _pd2 | 0,0574727 | .0121898 | 4.71 | 0.000 | .0335812 | .0813641 |
| _pd3 | 0,2763726 | .0142008 | 19.46 | 0.000 | .2485394 | .3042057 |
| _ad2 | 0,0956366 | .0126401 | 7.57 | 0.000 | .0708626 | .1204107 |
| _ad3 | 0,3329775 | .0143025 | 23.28 | 0.000 | .3049452 | .3610098 |
| _cons | -0,0020581 | .0091395 | -0.23 | 0.822 | -.0199713 | .015855 |
