# Supplementary material for: Valuing health-related quality of life using a hybrid approach: Tunisian value set for the EQ-5D-3L
Source: Qual Life Res. 2021 Jan 14;30(5):1445–55. doi: 10.1007/s11136-020-02730-z (PMC8068700; doi:10.1007/s11136-020-02730-z)
Supplement: Supplementary file 2 — (DOCX 21 kb) [file 11136_2020_2730_MOESM2_ESM.docx]

Tunisian value set for the EQ-5D-3L health states

| Health state | Utility value | Health state | Utility value | Health state | Utility value |
| --- | --- | --- | --- | --- | --- |
| 11111 | 1 | **21111** | 0,9243998 | **31111** | 0,4026229 |
| 11112 | 0,9052333 | **21112** | 0,8296331 | **31112** | 0,3078562 |
| 11113 | 0,6677436 | **21113** | 0,5921434 | **31113** | 0,0703665 |
| 11121 | 0,943234 | **21121** | 0,8676338 | **31121** | 0,3458569 |
| 11122 | 0,8484673 | **21122** | 0,7728671 | **31122** | 0,2510902 |
| 11123 | 0,6109776 | **21123** | 0,5353774 | **31123** | 0,0136005 |
| 11131 | 0,723979 | **21131** | 0,6483788 | **31131** | 0,1266019 |
| 11132 | 0,6292123 | **21132** | 0,5536121 | **31132** | 0,0318352 |
| 11133 | 0,3917226 | **21133** | 0,3161224 | **31133** | -0,2056545 |
| 11211 | 0,9218315 | **21211** | 0,8462313 | **31211** | 0,3244544 |
| 11212 | 0,8270648 | **21212** | 0,7514646 | **31212** | 0,2296877 |
| 11213 | 0,5895751 | **21213** | 0,5139749 | **31213** | -0,007802 |
| 11221 | 0,8650655 | **21221** | 0,7894653 | **31221** | 0,2676884 |
| 11222 | 0,7702988 | **21222** | 0,6946986 | **31222** | 0,1729217 |
| 11223 | 0,5328091 | **21223** | 0,4572089 | **31223** | -0,064568 |
| 11231 | 0,6458105 | **21231** | 0,5702103 | **31231** | 0,0484334 |
| 11232 | 0,5510438 | **21232** | 0,4754436 | **31232** | -0,0463333 |
| 11233 | 0,3135541 | **21233** | 0,2379539 | **31233** | -0,283823 |
| 11311 | 0,7493996 | **21311** | 0,6737994 | **31311** | 0,1520225 |
| 11312 | 0,6546329 | **21312** | 0,5790327 | **31312** | 0,0572558 |
| 11313 | 0,4171432 | **21313** | 0,341543 | **31313** | -0,1802339 |
| 11321 | 0,6926336 | **21321** | 0,6170334 | **31321** | 0,0952565 |
| 11322 | 0,5978669 | **21322** | 0,5222667 | **31322** | 0,0004898 |
| 11323 | 0,3603772 | **21323** | 0,284777 | **31323** | -0,2369999 |
| 11331 | 0,4733786 | **21331** | 0,3977784 | **31331** | -0,1239985 |
| 11332 | 0,3786119 | **21332** | 0,3030117 | **31332** | -0,2187652 |
| 11333 | 0,1411222 | **21333** | 0,065522 | **31333** | -0,4562549 |
| 12111 | 0,8348644 | **22111** | 0,7592642 | **32111** | 0,2374873 |
| 12112 | 0,7400977 | **22112** | 0,6644975 | **32112** | 0,1427206 |
| 12113 | 0,502608 | **22113** | 0,4270078 | **32113** | -0,0947691 |
| 12121 | 0,7780984 | **22121** | 0,7024982 | **32121** | 0,1807213 |
| 12122 | 0,6833317 | **22122** | 0,6077315 | **32122** | 0,0859546 |
| 12123 | 0,445842 | **22123** | 0,3702418 | **32123** | -0,1515351 |
| 12131 | 0,5588434 | **22131** | 0,4832432 | **32131** | -0,0385337 |
| 12132 | 0,4640767 | **22132** | 0,3884765 | **32132** | -0,1333004 |
| 12133 | 0,226587 | **22133** | 0,1509868 | **32133** | -0,3707901 |
| 12211 | 0,7566959 | **22211** | 0,6810957 | **32211** | 0,1593188 |
| 12212 | 0,6619292 | **22212** | 0,586329 | **32212** | 0,0645521 |
| 12213 | 0,4244395 | **22213** | 0,3488393 | **32213** | -0,1729376 |
| 12221 | 0,6999299 | **22221** | 0,6243297 | **32221** | 0,1025528 |
| 12222 | 0,6051632 | **22222** | 0,529563 | **32222** | 0,0077861 |
| 12223 | 0,3676735 | **22223** | 0,2920733 | **32223** | -0,2297036 |
| 12231 | 0,4806749 | **22231** | 0,4050747 | **32231** | -0,1167022 |
| 12232 | 0,3859082 | **22232** | 0,310308 | **32232** | -0,2114689 |
| 12233 | 0,1484185 | **22233** | 0,0728183 | **32233** | -0,4489586 |
| 12311 | 0,584264 | **22311** | 0,5086638 | **32311** | -0,0131131 |
| 12312 | 0,4894973 | **22312** | 0,4138971 | **32312** | -0,1078798 |
| 12313 | 0,2520076 | **22313** | 0,1764074 | **32313** | -0,3453695 |
| 12321 | 0,527498 | **22321** | 0,4518978 | **32321** | -0,0698791 |
| 12322 | 0,4327313 | **22322** | 0,3571311 | **32322** | -0,1646458 |
| 12323 | 0,1952416 | **22323** | 0,1196414 | **32323** | -0,4021355 |
| 12331 | 0,308243 | **22331** | 0,2326428 | **32331** | -0,2891341 |
| 12332 | 0,2134763 | **22332** | 0,1378761 | **32332** | -0,3839008 |
| 12333 | -0,0240134 | **22333** | -0,0996136 | **32333** | -0,6213905 |
| 13111 | 0,6636872 | **23111** | 0,588087 | **33111** | 0,0663101 |
| 13112 | 0,5689205 | **23112** | 0,4933203 | **33112** | -0,0284566 |
| 13113 | 0,3314308 | **23113** | 0,2558306 | **33113** | -0,2659463 |
| 13121 | 0,6069212 | **23121** | 0,531321 | **33121** | 0,0095441 |
| 13122 | 0,5121545 | **23122** | 0,4365543 | **33122** | -0,0852226 |
| 13123 | 0,2746648 | **23123** | 0,1990646 | **33123** | -0,3227123 |
| 13131 | 0,3876662 | **23131** | 0,312066 | **33131** | -0,2097109 |
| 13132 | 0,2928995 | **23132** | 0,2172993 | **33132** | -0,3044776 |
| 13133 | 0,0554098 | **23133** | -0,0201904 | **33133** | -0,5419673 |
| 13211 | 0,5855187 | **23211** | 0,5099185 | **33211** | -0,0118584 |
| 13212 | 0,490752 | **23212** | 0,4151518 | **33212** | -0,1066251 |
| 13213 | 0,2532623 | **23213** | 0,1776621 | **33213** | -0,3441148 |
| 13221 | 0,5287527 | **23221** | 0,4531525 | **33221** | -0,0686244 |
| 13222 | 0,433986 | **23222** | 0,3583858 | **33222** | -0,1633911 |
| 13223 | 0,1964963 | **23223** | 0,1208961 | **33223** | -0,4008808 |
| 13231 | 0,3094977 | **23231** | 0,2338975 | **33231** | -0,2878794 |
| 13232 | 0,214731 | **23232** | 0,1391308 | **33232** | -0,3826461 |
| 13233 | -0,0227587 | **23233** | -0,0983589 | **33233** | -0,6201358 |
| 13311 | 0,4130868 | **23311** | 0,3374866 | **33311** | -0,1842903 |
| 13312 | 0,3183201 | **23312** | 0,2427199 | **33312** | -0,279057 |
| 13313 | 0,0808304 | **23313** | 0,0052302 | **33313** | -0,5165467 |
| 13321 | 0,3563208 | **23321** | 0,2807206 | **33321** | -0,2410563 |
| 13322 | 0,2615541 | **23322** | 0,1859539 | **33322** | -0,335823 |
| 13323 | 0,0240644 | **23323** | -0,0515358 | **33323** | -0,5733127 |
| 13331 | 0,1370658 | **23331** | 0,0614656 | **33331** | -0,4603113 |
| 13332 | 0,0422991 | **23332** | -0,0333011 | **33332** | -0,555078 |
| 13333 | -0,1951906 | **23333** | -0,2707908 | **33333** | -0,7925677 |
