# Supplementary material for: Valuing health-related quality of life using a hybrid approach: Tunisian value set for the EQ-5D-3L
Source: Qual Life Res. 2021 Jan 14;30(5):1445–55. doi: 10.1007/s11136-020-02730-z (PMC8068700; doi:10.1007/s11136-020-02730-z)
Supplement: Supplementary file 3 — (DOCX 79 kb) [file 11136_2020_2730_MOESM3_ESM.docx]

**EQ-5D-5L Tunisian crosswalk value set**

| Health state | Utility | Health state | Utility | Health state | Utility | Health state | Utility |
| --- | --- | --- | --- | --- | --- | --- | --- |
| 11111 | 1 | 22222 | 0,6169365 | 33333 | 0,529563 | 44444 | 0,18957321 |
| 11112 | 0,9248125 | 22223 | 0,5973574 | 33334 | 0,4130308 | 44445 | 0,06861572 |
| 11113 | 0,9052333 | 22224 | 0,4808252 | 33335 | 0,2920733 | 44451 | 0,26920894 |
| 11114 | 0,7887011 | 22225 | 0,3598677 | 33341 | 0,5367379 | 44452 | 0,1940214 |
| 11115 | 0,6677436 | 22231 | 0,6808351 | 33342 | 0,4615503 | 44453 | 0,17444224 |
| 11121 | 0,954523 | 22232 | 0,6056475 | 33343 | 0,4419712 | 44454 | 0,05791003 |
| 11122 | 0,8793355 | 22233 | 0,5860684 | 33344 | 0,325439 | 44455 | -0,06304746 |
| 11123 | 0,8597563 | 22234 | 0,4695362 | 33345 | 0,2044815 | 44511 | 0,42943009 |
| 11124 | 0,7432241 | 22235 | 0,3485787 | 33351 | 0,4050747 | 44512 | 0,35424255 |
| 11125 | 0,6222666 | 22241 | 0,5932432 | 33352 | 0,3298872 | 44513 | 0,33466339 |
| 11131 | 0,943234 | 22242 | 0,5180557 | 33353 | 0,310308 | 44514 | 0,21813118 |
| 11132 | 0,8680465 | 22243 | 0,4984765 | 33354 | 0,1937758 | 44515 | 0,09717369 |
| 11133 | 0,8484673 | 22244 | 0,3819443 | 33355 | 0,0728183 | 44521 | 0,38395309 |
| 11134 | 0,7319351 | 22245 | 0,2609868 | 33411 | 0,6244637 | 44522 | 0,30876554 |
| 11135 | 0,6109776 | 22251 | 0,4615801 | 33412 | 0,5492761 | 44523 | 0,28918639 |
| 11141 | 0,8556422 | 22252 | 0,3863925 | 33413 | 0,529697 | 44524 | 0,17265417 |
| 11142 | 0,7804546 | 22253 | 0,3668134 | 33414 | 0,4131647 | 44525 | 0,05169669 |
| 11143 | 0,7608755 | 22254 | 0,2502812 | 33415 | 0,2922073 | 44531 | 0,37266409 |
| 11144 | 0,6443433 | 22255 | 0,1293237 | 33421 | 0,5789867 | 44532 | 0,29747655 |
| 11145 | 0,5233858 | 22311 | 0,7221381 | 33422 | 0,5037991 | 44533 | 0,27789739 |
| 11151 | 0,723979 | 22312 | 0,6469506 | 33423 | 0,48422 | 44534 | 0,16136518 |
| 11152 | 0,6487915 | 22313 | 0,6273714 | 33424 | 0,3676877 | 44535 | 0,04040769 |
| 11153 | 0,6292123 | 22314 | 0,5108392 | 33425 | 0,2467303 | 44541 | 0,28507227 |
| 11154 | 0,5126801 | 22315 | 0,3898817 | 33431 | 0,5676977 | 44542 | 0,20988472 |
| 11155 | 0,3917226 | 22321 | 0,6766611 | 33432 | 0,4925101 | 44543 | 0,19030557 |
| 11211 | 0,9372944 | 22322 | 0,6014736 | 33433 | 0,472931 | 44544 | 0,07377335 |
| 11212 | 0,8621069 | 22323 | 0,5818944 | 33434 | 0,3563987 | 44545 | -0,04718413 |
| 11213 | 0,8425277 | 22324 | 0,4653622 | 33435 | 0,2354413 | 44551 | 0,15340909 |
| 11214 | 0,7259955 | 22325 | 0,3444047 | 33441 | 0,4801058 | 44552 | 0,07822155 |
| 11215 | 0,605038 | 22331 | 0,6653721 | 33442 | 0,4049183 | 44553 | 0,05864239 |
| 11221 | 0,8918174 | 22332 | 0,5901846 | 33443 | 0,3853391 | 44554 | -0,05788982 |
| 11222 | 0,8166299 | 22333 | 0,5706054 | 33444 | 0,2688069 | 44555 | -0,17884731 |
| 11223 | 0,7970507 | 22334 | 0,4540732 | 33445 | 0,1478494 | 45111 | 0,55045886 |
| 11224 | 0,6805185 | 22335 | 0,3331157 | 33451 | 0,3484427 | 45112 | 0,47527132 |
| 11225 | 0,559561 | 22341 | 0,5777803 | 33452 | 0,2732551 | 45113 | 0,45569216 |
| 11231 | 0,8805284 | 22342 | 0,5025928 | 33453 | 0,253676 | 45114 | 0,33915995 |
| 11232 | 0,8053409 | 22343 | 0,4830136 | 33454 | 0,1371437 | 45115 | 0,21820246 |
| 11233 | 0,7857617 | 22344 | 0,3664814 | 33455 | 0,0161863 | 45121 | 0,50498186 |
| 11234 | 0,6692295 | 22345 | 0,2455239 | 33511 | 0,5086638 | 45122 | 0,42979431 |
| 11235 | 0,548272 | 22351 | 0,4461171 | 33512 | 0,4334763 | 45123 | 0,41021516 |
| 11241 | 0,7929366 | 22352 | 0,3709296 | 33513 | 0,4138971 | 45124 | 0,29368294 |
| 11242 | 0,7177491 | 22353 | 0,3513504 | 33514 | 0,2973649 | 45125 | 0,17272546 |
| 11243 | 0,6981699 | 22354 | 0,2348182 | 33515 | 0,1764074 | 45131 | 0,49369286 |
| 11244 | 0,5816377 | 22355 | 0,1138607 | 33521 | 0,4631868 | 45132 | 0,41850532 |
| 11245 | 0,4606802 | 22411 | 0,6655061 | 33522 | 0,3879993 | 45133 | 0,39892616 |
| 11251 | 0,6612734 | 22412 | 0,5903185 | 33523 | 0,3684201 | 45134 | 0,28239395 |
| 11252 | 0,5860859 | 22413 | 0,5707394 | 33524 | 0,2518879 | 45135 | 0,16143646 |
| 11253 | 0,5665067 | 22414 | 0,4542072 | 33525 | 0,1309304 | 45141 | 0,40610104 |
| 11254 | 0,4499745 | 22415 | 0,3332497 | 33531 | 0,4518978 | 45142 | 0,33091349 |
| 11255 | 0,329017 | 22421 | 0,6200291 | 33532 | 0,3767103 | 45143 | 0,31133434 |
| 11311 | 0,9218315 | 22422 | 0,5448415 | 33533 | 0,3571311 | 45144 | 0,19480212 |
| 11312 | 0,846644 | 22423 | 0,5252624 | 33534 | 0,2405989 | 45145 | 0,07384464 |
| 11313 | 0,8270648 | 22424 | 0,4087302 | 33535 | 0,1196414 | 45151 | 0,27443786 |
| 11314 | 0,7105326 | 22425 | 0,2877727 | 33541 | 0,364306 | 45152 | 0,19925032 |
| 11315 | 0,5895751 | 22431 | 0,6087401 | 33542 | 0,2891184 | 45153 | 0,17967116 |
| 11321 | 0,8763545 | 22432 | 0,5335525 | 33543 | 0,2695393 | 45154 | 0,06313895 |
| 11322 | 0,801167 | 22433 | 0,5139734 | 33544 | 0,1530071 | 45155 | -0,0578185 |
| 11323 | 0,7815878 | 22434 | 0,3974412 | 33545 | 0,0320496 | 45211 | 0,4877533 |
| 11324 | 0,6650556 | 22435 | 0,2764837 | 33551 | 0,2326428 | 45212 | 0,41256576 |
| 11325 | 0,5440981 | 22441 | 0,5211483 | 33552 | 0,1574553 | 45213 | 0,3929866 |
| 11331 | 0,8650655 | 22442 | 0,4459607 | 33553 | 0,1378761 | 45214 | 0,27645439 |
| 11332 | 0,789878 | 22443 | 0,4263816 | 33554 | 0,0213439 | 45215 | 0,1554969 |
| 11333 | 0,7702988 | 22444 | 0,3098493 | 33555 | -0,099613 | 45221 | 0,4422763 |
| 11334 | 0,6537666 | 22445 | 0,1888919 | 34111 | 0,7176586 | 45222 | 0,36708876 |
| 11335 | 0,5328091 | 22451 | 0,3894851 | 34112 | 0,6424711 | 45223 | 0,3475096 |
| 11341 | 0,7774737 | 22452 | 0,3142975 | 34113 | 0,6228919 | 45224 | 0,23097739 |
| 11342 | 0,7022861 | 22453 | 0,2947184 | 34114 | 0,5063597 | 45225 | 0,1100199 |
| 11343 | 0,682707 | 22454 | 0,1781862 | 34115 | 0,3854022 | 45231 | 0,4309873 |
| 11344 | 0,5661748 | 22455 | 0,0572287 | 34121 | 0,6721816 | 45232 | 0,35579976 |
| 11345 | 0,4452173 | 22511 | 0,5497062 | 34122 | 0,5969941 | 45233 | 0,3362206 |
| 11351 | 0,6458105 | 22512 | 0,4745187 | 34123 | 0,5774149 | 45234 | 0,21968839 |
| 11352 | 0,570623 | 22513 | 0,4549395 | 34124 | 0,4608827 | 45235 | 0,0987309 |
| 11353 | 0,5510438 | 22514 | 0,3384073 | 34125 | 0,3399252 | 45241 | 0,34339548 |
| 11354 | 0,4345116 | 22515 | 0,2174498 | 34131 | 0,6608926 | 45242 | 0,26820794 |
| 11355 | 0,3135541 | 22521 | 0,5042292 | 34132 | 0,5857051 | 45243 | 0,24862878 |
| 11411 | 0,8651995 | 22522 | 0,4290417 | 34133 | 0,5661259 | 45244 | 0,13209657 |
| 11412 | 0,7900119 | 22523 | 0,4094625 | 34134 | 0,4495937 | 45245 | 0,01113908 |
| 11413 | 0,7704328 | 22524 | 0,2929303 | 34135 | 0,3286362 | 45251 | 0,2117323 |
| 11414 | 0,6539005 | 22525 | 0,1719728 | 34141 | 0,5733008 | 45252 | 0,13654476 |
| 11415 | 0,5329431 | 22531 | 0,4929402 | 34142 | 0,4981133 | 45253 | 0,1169656 |
| 11421 | 0,8197225 | 22532 | 0,4177527 | 34143 | 0,4785341 | 45254 | 0,00043339 |
| 11422 | 0,7445349 | 22533 | 0,3981735 | 34144 | 0,3620019 | 45255 | -0,1205241 |
| 11423 | 0,7249558 | 22534 | 0,2816413 | 34145 | 0,2410444 | 45311 | 0,47229036 |
| 11424 | 0,6084235 | 22535 | 0,1606838 | 34151 | 0,4416376 | 45312 | 0,39710282 |
| 11425 | 0,4874661 | 22541 | 0,4053484 | 34152 | 0,3664501 | 45313 | 0,37752366 |
| 11431 | 0,8084335 | 22542 | 0,3301609 | 34153 | 0,3468709 | 45314 | 0,26099145 |
| 11432 | 0,7332459 | 22543 | 0,3105817 | 34154 | 0,2303387 | 45315 | 0,14003396 |
| 11433 | 0,7136668 | 22544 | 0,1940495 | 34155 | 0,1093812 | 45321 | 0,42681336 |
| 11434 | 0,5971345 | 22545 | 0,073092 | 34211 | 0,6549531 | 45322 | 0,35162581 |
| 11435 | 0,4761771 | 22551 | 0,2736852 | 34212 | 0,5797655 | 45323 | 0,33204666 |
| 11441 | 0,7208416 | 22552 | 0,1984977 | 34213 | 0,5601864 | 45324 | 0,21551444 |
| 11442 | 0,6456541 | 22553 | 0,1789185 | 34214 | 0,4436542 | 45325 | 0,09455696 |
| 11443 | 0,6260749 | 22554 | 0,0623863 | 34215 | 0,3226967 | 45331 | 0,41552436 |
| 11444 | 0,5095427 | 22555 | -0,058571 | 34221 | 0,6094761 | 45332 | 0,34033682 |
| 11445 | 0,3885852 | 23111 | 0,7726717 | 34222 | 0,5342885 | 45333 | 0,32075766 |
| 11451 | 0,5891785 | 23112 | 0,6974841 | 34223 | 0,5147094 | 45334 | 0,20422545 |
| 11452 | 0,5139909 | 23113 | 0,677905 | 34224 | 0,3981772 | 45335 | 0,08326796 |
| 11453 | 0,4944118 | 23114 | 0,5613728 | 34225 | 0,2772197 | 45341 | 0,32793254 |
| 11454 | 0,3778795 | 23115 | 0,4404153 | 34231 | 0,5981871 | 45342 | 0,25274499 |
| 11455 | 0,2569221 | 23121 | 0,7271947 | 34232 | 0,5229995 | 45343 | 0,23316584 |
| 11511 | 0,7493996 | 23122 | 0,6520071 | 34233 | 0,5034204 | 45344 | 0,11663362 |
| 11512 | 0,6742121 | 23123 | 0,632428 | 34234 | 0,3868882 | 45345 | -0,0043238 |
| 11513 | 0,6546329 | 23124 | 0,5158958 | 34235 | 0,2659307 | 45351 | 0,19626936 |
| 11514 | 0,5381007 | 23125 | 0,3949383 | 34241 | 0,5105953 | 45352 | 0,12108182 |
| 11515 | 0,4171432 | 23131 | 0,7159057 | 34242 | 0,4354077 | 45353 | 0,10150266 |
| 11521 | 0,7039226 | 23132 | 0,6407181 | 34243 | 0,4158286 | 45354 | -0,0150295 |
| 11522 | 0,6287351 | 23133 | 0,621139 | 34244 | 0,2992963 | 45355 | -0,1359870 |
| 11523 | 0,6091559 | 23134 | 0,5046068 | 34245 | 0,1783389 | 45411 | 0,41565831 |
| 11524 | 0,4926237 | 23135 | 0,3836493 | 34251 | 0,3789321 | 45412 | 0,34047077 |
| 11525 | 0,3716662 | 23141 | 0,6283139 | 34252 | 0,3037445 | 45413 | 0,32089161 |
| 11531 | 0,6926336 | 23142 | 0,5531263 | 34253 | 0,2841654 | 45414 | 0,2043594 |
| 11532 | 0,6174461 | 23143 | 0,5335472 | 34254 | 0,1676332 | 45415 | 0,08340191 |
| 11533 | 0,5978669 | 23144 | 0,417015 | 34255 | 0,0466757 | 45421 | 0,37018131 |
| 11534 | 0,4813347 | 23145 | 0,2960575 | 34311 | 0,6394901 | 45422 | 0,29499377 |
| 11535 | 0,3603772 | 23151 | 0,4966507 | 34312 | 0,5643026 | 45423 | 0,27541461 |
| 11541 | 0,6050418 | 23152 | 0,4214631 | 34313 | 0,5447234 | 45424 | 0,1588824 |
| 11542 | 0,5298542 | 23153 | 0,401884 | 34314 | 0,4281912 | 45425 | 0,03792491 |
| 11543 | 0,5102751 | 23154 | 0,2853518 | 34315 | 0,3072337 | 45431 | 0,35889231 |
| 11544 | 0,3937429 | 23155 | 0,1643943 | 34321 | 0,5940131 | 45432 | 0,28370477 |
| 11545 | 0,2727854 | 23211 | 0,7099661 | 34322 | 0,5188256 | 45433 | 0,26412561 |
| 11551 | 0,4733786 | 23212 | 0,6347786 | 34323 | 0,4992464 | 45434 | 0,1475934 |
| 11552 | 0,3981911 | 23213 | 0,6151994 | 34324 | 0,3827142 | 45435 | 0,02663591 |
| 11553 | 0,3786119 | 23214 | 0,4986672 | 34325 | 0,2617567 | 45441 | 0,27130049 |
| 11554 | 0,2620797 | 23215 | 0,3777097 | 34331 | 0,5827241 | 45442 | 0,19611295 |
| 11555 | 0,1411222 | 23221 | 0,6644891 | 34332 | 0,5075366 | 45443 | 0,17653379 |
| 12111 | 0,8624993 | 23222 | 0,5893016 | 34333 | 0,4879574 | 45444 | 0,06000158 |
| 12112 | 0,7873118 | 23223 | 0,5697224 | 34334 | 0,3714252 | 45445 | -0,0609559 |
| 12113 | 0,7677326 | 23224 | 0,4531902 | 34335 | 0,2504677 | 45451 | 0,13963731 |
| 12114 | 0,6512004 | 23225 | 0,3322327 | 34341 | 0,4951323 | 45452 | 0,06444977 |
| 12115 | 0,5302429 | 23231 | 0,6532001 | 34342 | 0,4199448 | 45453 | 0,04487061 |
| 12121 | 0,8170223 | 23232 | 0,5780126 | 34343 | 0,4003656 | 45454 | -0,0716616 |
| 12122 | 0,7418348 | 23233 | 0,5584334 | 34344 | 0,2838334 | 45455 | -0,1926190 |
| 12123 | 0,7222556 | 23234 | 0,4419012 | 34345 | 0,1628759 | 45511 | 0,29985846 |
| 12124 | 0,6057234 | 23235 | 0,3209437 | 34351 | 0,3634691 | 45512 | 0,22467092 |
| 12125 | 0,4847659 | 23241 | 0,5656083 | 34352 | 0,2882816 | 45513 | 0,20509176 |
| 12131 | 0,8057333 | 23242 | 0,4904208 | 34353 | 0,2687024 | 45514 | 0,08855955 |
| 12132 | 0,7305458 | 23243 | 0,4708416 | 34354 | 0,1521702 | 45515 | -0,0323979 |
| 12133 | 0,7109666 | 23244 | 0,3543094 | 34355 | 0,0312127 | 45521 | 0,25438146 |
| 12134 | 0,5944344 | 23245 | 0,2333519 | 34411 | 0,5828581 | 45522 | 0,17919391 |
| 12135 | 0,4734769 | 23251 | 0,4339451 | 34412 | 0,5076705 | 45523 | 0,15961476 |
| 12141 | 0,7181415 | 23252 | 0,3587576 | 34413 | 0,4880914 | 45524 | 0,04308254 |
| 12142 | 0,642954 | 23253 | 0,3391784 | 34414 | 0,3715592 | 45525 | -0,0778749 |
| 12143 | 0,6233748 | 23254 | 0,2226462 | 34415 | 0,2506017 | 45531 | 0,24309246 |
| 12144 | 0,5068426 | 23255 | 0,1016887 | 34421 | 0,5373811 | 45532 | 0,16790492 |
| 12145 | 0,3858851 | 23311 | 0,6945032 | 34422 | 0,4621935 | 45533 | 0,14832576 |
| 12151 | 0,5864783 | 23312 | 0,6193156 | 34423 | 0,4426144 | 45534 | 0,03179355 |
| 12152 | 0,5112908 | 23313 | 0,5997365 | 34424 | 0,3260822 | 45535 | -0,0891639 |
| 12153 | 0,4917116 | 23314 | 0,4832043 | 34425 | 0,2051247 | 45541 | 0,15550064 |
| 12154 | 0,3751794 | 23315 | 0,3622468 | 34431 | 0,5260921 | 45542 | 0,08031309 |
| 12155 | 0,2542219 | 23321 | 0,6490262 | 34432 | 0,4509045 | 45543 | 0,06073394 |
| 12211 | 0,7997938 | 23322 | 0,5738386 | 34433 | 0,4313254 | 45544 | -0,0557982 |
| 12212 | 0,7246062 | 23323 | 0,5542595 | 34434 | 0,3147932 | 45545 | -0,1767557 |
| 12213 | 0,7050271 | 23324 | 0,4377273 | 34435 | 0,1938357 | 45551 | 0,02383746 |
| 12214 | 0,5884949 | 23325 | 0,3167698 | 34441 | 0,4385003 | 45552 | -0,0513500 |
| 12215 | 0,4675374 | 23331 | 0,6377372 | 34442 | 0,3633127 | 45553 | -0,0709292 |
| 12221 | 0,7543168 | 23332 | 0,5625496 | 34443 | 0,3437336 | 45554 | -0,1874614 |
| 12222 | 0,6791292 | 23333 | 0,5429705 | 34444 | 0,2272014 | 45555 | -0,3084189 |
| 12223 | 0,6595501 | 23334 | 0,4264383 | 34445 | 0,1062439 | 51111 | 0,4026229 |
| 12224 | 0,5430179 | 23335 | 0,3054808 | 34451 | 0,3068371 | 51112 | 0,32743536 |
| 12225 | 0,4220604 | 23341 | 0,5501454 | 34452 | 0,2316495 | 51113 | 0,3078562 |
| 12231 | 0,7430278 | 23342 | 0,4749578 | 34453 | 0,2120704 | 51114 | 0,19132399 |
| 12232 | 0,6678402 | 23343 | 0,4553787 | 34454 | 0,0955382 | 51115 | 0,0703665 |
| 12233 | 0,6482611 | 23344 | 0,3388465 | 34455 | -0,025419 | 51121 | 0,3571459 |
| 12234 | 0,5317289 | 23345 | 0,217889 | 34511 | 0,4670582 | 51122 | 0,28195836 |
| 12235 | 0,4107714 | 23351 | 0,4184822 | 34512 | 0,3918707 | 51123 | 0,2623792 |
| 12241 | 0,655436 | 23352 | 0,3432946 | 34513 | 0,3722915 | 51124 | 0,14584698 |
| 12242 | 0,5802484 | 23353 | 0,3237155 | 34514 | 0,2557593 | 51125 | 0,0248895 |
| 12243 | 0,5606693 | 23354 | 0,2071833 | 34515 | 0,1348018 | 51131 | 0,3458569 |
| 12244 | 0,444137 | 23355 | 0,0862258 | 34521 | 0,4215812 | 51132 | 0,27066936 |
| 12245 | 0,3231796 | 23411 | 0,6378711 | 34522 | 0,3463937 | 51133 | 0,2510902 |
| 12251 | 0,5237728 | 23412 | 0,5626836 | 34523 | 0,3268145 | 51134 | 0,13455799 |
| 12252 | 0,4485852 | 23413 | 0,5431044 | 34524 | 0,2102823 | 51135 | 0,0136005 |
| 12253 | 0,4290061 | 23414 | 0,4265722 | 34525 | 0,0893248 | 51141 | 0,25826508 |
| 12254 | 0,3124739 | 23415 | 0,3056147 | 34531 | 0,4102922 | 51142 | 0,18307754 |
| 12255 | 0,1915164 | 23421 | 0,5923941 | 34532 | 0,3351047 | 51143 | 0,16349838 |
| 12311 | 0,7843308 | 23422 | 0,5172066 | 34533 | 0,3155255 | 51144 | 0,04696617 |
| 12312 | 0,7091433 | 23423 | 0,4976274 | 34534 | 0,1989933 | 51145 | -0,0739913 |
| 12313 | 0,6895641 | 23424 | 0,3810952 | 34535 | 0,0780358 | 51151 | 0,1266019 |
| 12314 | 0,5730319 | 23425 | 0,2601377 | 34541 | 0,3227004 | 51152 | 0,05141436 |
| 12315 | 0,4520744 | 23431 | 0,5811051 | 34542 | 0,2475129 | 51153 | 0,0318352 |
| 12321 | 0,7388538 | 23432 | 0,5059176 | 34543 | 0,2279337 | 51154 | -0,0846970 |
| 12322 | 0,6636663 | 23433 | 0,4863384 | 34544 | 0,1114015 | 51155 | -0,2056545 |
| 12323 | 0,6440871 | 23434 | 0,3698062 | 34545 | -0,009556 | 51211 | 0,33991734 |
| 12324 | 0,5275549 | 23435 | 0,2488487 | 34551 | 0,1910372 | 51212 | 0,2647298 |
| 12325 | 0,4065974 | 23441 | 0,4935133 | 34552 | 0,1158497 | 51213 | 0,24515064 |
| 12331 | 0,7275648 | 23442 | 0,4183258 | 34553 | 0,0962705 | 51214 | 0,12861843 |
| 12332 | 0,6523773 | 23443 | 0,3987466 | 34554 | -0,020261 | 51215 | 0,00766094 |
| 12333 | 0,6327981 | 23444 | 0,2822144 | 34555 | -0,141219 | 51221 | 0,29444034 |
| 12334 | 0,5162659 | 23445 | 0,1612569 | 35111 | 0,588087 | 51222 | 0,2192528 |
| 12335 | 0,3953084 | 23451 | 0,3618501 | 35112 | 0,5128995 | 51223 | 0,19967364 |
| 12341 | 0,639973 | 23452 | 0,2866626 | 35113 | 0,4933203 | 51224 | 0,08314143 |
| 12342 | 0,5647855 | 23453 | 0,2670834 | 35114 | 0,3767881 | 51225 | -0,0378160 |
| 12343 | 0,5452063 | 23454 | 0,1505512 | 35115 | 0,2558306 | 51231 | 0,28315134 |
| 12344 | 0,4286741 | 23455 | 0,0295937 | 35121 | 0,54261 | 51232 | 0,2079638 |
| 12345 | 0,3077166 | 23511 | 0,5220713 | 35122 | 0,4674225 | 51233 | 0,18838464 |
| 12351 | 0,5083098 | 23512 | 0,4468837 | 35123 | 0,4478433 | 51234 | 0,07185243 |
| 12352 | 0,4331223 | 23513 | 0,4273046 | 35124 | 0,3313111 | 51235 | -0,0491050 |
| 12353 | 0,4135431 | 23514 | 0,3107724 | 35125 | 0,2103536 | 51241 | 0,19555952 |
| 12354 | 0,2970109 | 23515 | 0,1898149 | 35131 | 0,531321 | 51242 | 0,12037198 |
| 12355 | 0,1760534 | 23521 | 0,4765943 | 35132 | 0,4561335 | 51243 | 0,10079282 |
| 12411 | 0,7276988 | 23522 | 0,4014067 | 35133 | 0,4365543 | 51244 | -0,0157393 |
| 12412 | 0,6525112 | 23523 | 0,3818276 | 35134 | 0,3200221 | 51245 | -0,1366968 |
| 12413 | 0,6329321 | 23524 | 0,2652954 | 35135 | 0,1990646 | 51251 | 0,06389634 |
| 12414 | 0,5163999 | 23525 | 0,1443379 | 35141 | 0,4437292 | 51252 | -0,0112912 |
| 12415 | 0,3954424 | 23531 | 0,4653053 | 35142 | 0,3685416 | 51253 | -0,0308703 |
| 12421 | 0,6822218 | 23532 | 0,3901177 | 35143 | 0,3489625 | 51254 | -0,1474025 |
| 12422 | 0,6070342 | 23533 | 0,3705386 | 35144 | 0,2324303 | 51255 | -0,2683600 |
| 12423 | 0,5874551 | 23534 | 0,2540064 | 35145 | 0,1114728 | 51311 | 0,3244544 |
| 12424 | 0,4709229 | 23535 | 0,1330489 | 35151 | 0,312066 | 51312 | 0,24926686 |
| 12425 | 0,3499654 | 23541 | 0,3777135 | 35152 | 0,2368785 | 51313 | 0,2296877 |
| 12431 | 0,6709328 | 23542 | 0,3025259 | 35153 | 0,2172993 | 51314 | 0,11315549 |
| 12432 | 0,5957452 | 23543 | 0,2829468 | 35154 | 0,1007671 | 51315 | -0,007802 |
| 12433 | 0,5761661 | 23544 | 0,1664146 | 35155 | -0,020190 | 51321 | 0,2789774 |
| 12434 | 0,4596339 | 23545 | 0,0454571 | 35211 | 0,5253814 | 51322 | 0,20378986 |
| 12435 | 0,3386764 | 23551 | 0,2460503 | 35212 | 0,4501939 | 51323 | 0,1842107 |
| 12441 | 0,583341 | 23552 | 0,1708627 | 35213 | 0,4306147 | 51324 | 0,06767848 |
| 12442 | 0,5081534 | 23553 | 0,1512836 | 35214 | 0,3140825 | 51325 | -0,053279 |
| 12443 | 0,4885743 | 23554 | 0,0347514 | 35215 | 0,193125 | 51331 | 0,2676884 |
| 12444 | 0,3720421 | 23555 | -0,086206 | 35221 | 0,4799044 | 51332 | 0,19250086 |
| 12445 | 0,2510846 | 24111 | 0,7310661 | 35222 | 0,4047169 | 51333 | 0,1729217 |
| 12451 | 0,4516778 | 24112 | 0,6558786 | 35223 | 0,3851377 | 51334 | 0,05638949 |
| 12452 | 0,3764902 | 24113 | 0,6362994 | 35224 | 0,2686055 | 51335 | -0,064568 |
| 12453 | 0,3569111 | 24114 | 0,5197672 | 35225 | 0,147648 | 51341 | 0,18009658 |
| 12454 | 0,2403789 | 24115 | 0,3988097 | 35231 | 0,4686154 | 51342 | 0,10490904 |
| 12455 | 0,1194214 | 24121 | 0,6855891 | 35232 | 0,3934279 | 51343 | 0,08532988 |
| 12511 | 0,6118989 | 24122 | 0,6104016 | 35233 | 0,3738487 | 51344 | -0,0312023 |
| 12512 | 0,5367114 | 24123 | 0,5908224 | 35234 | 0,2573165 | 51345 | -0,1521598 |
| 12513 | 0,5171322 | 24124 | 0,4742902 | 35235 | 0,136359 | 51351 | 0,0484334 |
| 12514 | 0,4006 | 24125 | 0,3533327 | 35241 | 0,3810236 | 51352 | -0,0267541 |
| 12515 | 0,2796425 | 24131 | 0,6743001 | 35242 | 0,3058361 | 51353 | -0,0463333 |
| 12521 | 0,5664219 | 24132 | 0,5991126 | 35243 | 0,2862569 | 51354 | -0,1628655 |
| 12522 | 0,4912344 | 24133 | 0,5795334 | 35244 | 0,1697247 | 51355 | -0,283823 |
| 12523 | 0,4716552 | 24134 | 0,4630012 | 35245 | 0,0487672 | 51411 | 0,26782235 |
| 12524 | 0,355123 | 24135 | 0,3420437 | 35251 | 0,2493604 | 51412 | 0,19263481 |
| 12525 | 0,2341655 | 24141 | 0,5867083 | 35252 | 0,1741729 | 51413 | 0,17305565 |
| 12531 | 0,5551329 | 24142 | 0,5115208 | 35253 | 0,1545937 | 51414 | 0,05652344 |
| 12532 | 0,4799454 | 24143 | 0,4919416 | 35254 | 0,0380615 | 51415 | -0,0644340 |
| 12533 | 0,4603662 | 24144 | 0,3754094 | 35255 | -0,082896 | 51421 | 0,22234535 |
| 12534 | 0,343834 | 24145 | 0,2544519 | 35311 | 0,5099185 | 51422 | 0,14715781 |
| 12535 | 0,2228765 | 24151 | 0,4550451 | 35312 | 0,434731 | 51423 | 0,12757865 |
| 12541 | 0,4675411 | 24152 | 0,3798576 | 35313 | 0,4151518 | 51424 | 0,01104644 |
| 12542 | 0,3923536 | 24153 | 0,3602784 | 35314 | 0,2986196 | 51425 | -0,1099110 |
| 12543 | 0,3727744 | 24154 | 0,2437462 | 35315 | 0,1776621 | 51431 | 0,21105635 |
| 12544 | 0,2562422 | 24155 | 0,1227887 | 35321 | 0,4644415 | 51432 | 0,13586881 |
| 12545 | 0,1352847 | 24211 | 0,6683606 | 35322 | 0,389254 | 51433 | 0,11628965 |
| 12551 | 0,3358779 | 24212 | 0,593173 | 35323 | 0,3696748 | 51434 | -0,0002425 |
| 12552 | 0,2606904 | 24213 | 0,5735939 | 35324 | 0,2531426 | 51435 | -0,12120005 |
| 12553 | 0,2411112 | 24214 | 0,4570616 | 35325 | 0,1321851 | 51441 | 0,12346453 |
| 12554 | 0,124579 | 24215 | 0,3361042 | 35331 | 0,4531525 | 51442 | 0,04827699 |
| 12555 | 0,0036215 | 24221 | 0,6228836 | 35332 | 0,377965 | 51443 | 0,02869783 |
| 13111 | 0,8348644 | 24222 | 0,547696 | 35333 | 0,3583858 | 51444 | -0,08783438 |
| 13112 | 0,7596769 | 24223 | 0,5281169 | 35334 | 0,2418536 | 51445 | -0,20879187 |
| 13113 | 0,7400977 | 24224 | 0,4115846 | 35335 | 0,1208961 | 51451 | -0,00819865 |
| 13114 | 0,6235655 | 24225 | 0,2906272 | 35341 | 0,3655607 | 51452 | -0,08338619 |
| 13115 | 0,502608 | 24231 | 0,6115946 | 35342 | 0,2903731 | 51453 | -0,10296535 |
| 13121 | 0,7893874 | 24232 | 0,536407 | 35343 | 0,270794 | 51454 | -0,21949756 |
| 13122 | 0,7141999 | 24233 | 0,5168279 | 35344 | 0,1542618 | 51455 | -0,34045505 |
| 13123 | 0,6946207 | 24234 | 0,4002956 | 35345 | 0,0333043 | 51511 | 0,1520225 |
| 13124 | 0,5780885 | 24235 | 0,2793382 | 35351 | 0,2338975 | 51512 | 0,07683496 |
| 13125 | 0,457131 | 24241 | 0,5240027 | 35352 | 0,15871 | 51513 | 0,0572558 |
| 13131 | 0,7780984 | 24242 | 0,4488152 | 35353 | 0,1391308 | 51514 | -0,05927641 |
| 13132 | 0,7029109 | 24243 | 0,429236 | 35354 | 0,0225986 | 51515 | -0,1802339 |
| 13133 | 0,6833317 | 24244 | 0,3127038 | 35355 | -0,0983589 | 51521 | 0,1065455 |
| 13134 | 0,5667995 | 24245 | 0,1917463 | 35411 | 0,4532865 | 51522 | 0,03135796 |
| 13135 | 0,445842 | 24251 | 0,3923396 | 35412 | 0,3780989 | 51523 | 0,0117788 |
| 13141 | 0,6905066 | 24252 | 0,317152 | 35413 | 0,3585198 | 51524 | -0,10475342 |
| 13142 | 0,615319 | 24253 | 0,2975729 | 35414 | 0,2419875 | 51525 | -0,2257109 |
| 13143 | 0,5957399 | 24254 | 0,1810406 | 35415 | 0,1210301 | 51531 | 0,0952565 |
| 13144 | 0,4792077 | 24255 | 0,0600832 | 35421 | 0,4078095 | 51532 | 0,02006896 |
| 13145 | 0,3582502 | 24311 | 0,6528976 | 35422 | 0,3326219 | 51533 | 0,0004898 |
| 13151 | 0,5588434 | 24312 | 0,5777101 | 35423 | 0,3130428 | 51534 | -0,11604241 |
| 13152 | 0,4836559 | 24313 | 0,5581309 | 35424 | 0,1965105 | 51535 | -0,2369999 |
| 13153 | 0,4640767 | 24314 | 0,4415987 | 35425 | 0,0755531 | 51541 | 0,00766468 |
| 13154 | 0,3475445 | 24315 | 0,3206412 | 35431 | 0,3965205 | 51542 | -0,06752286 |
| 13155 | 0,226587 | 24321 | 0,6074206 | 35432 | 0,3213329 | 51543 | -0,08710202 |
| 13211 | 0,7721588 | 24322 | 0,5322331 | 35433 | 0,3017538 | 51544 | -0,20363423 |
| 13212 | 0,6969713 | 24323 | 0,5126539 | 35434 | 0,1852215 | 51545 | -0,32459172 |
| 13213 | 0,6773921 | 24324 | 0,3961217 | 35435 | 0,0642641 | 51551 | -0,1239985 |
| 13214 | 0,5608599 | 24325 | 0,2751642 | 35441 | 0,3089286 | 51552 | -0,19918604 |
| 13215 | 0,4399024 | 24331 | 0,5961316 | 35442 | 0,2337411 | 51553 | -0,2187652 |
| 13221 | 0,7266818 | 24332 | 0,5209441 | 35443 | 0,2141619 | 51554 | -0,33529741 |
| 13222 | 0,6514943 | 24333 | 0,5013649 | 35444 | 0,0976297 | 51555 | -0,4562549 |
| 13223 | 0,6319151 | 24334 | 0,3848327 | 35445 | -0,0233278 | 52111 | 0,26512224 |
| 13224 | 0,5153829 | 24335 | 0,2638752 | 35451 | 0,1772655 | 52112 | 0,18993469 |
| 13225 | 0,3944254 | 24341 | 0,5085398 | 35452 | 0,1020779 | 52113 | 0,17035554 |
| 13231 | 0,7153928 | 24342 | 0,4333523 | 35453 | 0,0824988 | 52114 | 0,05382332 |
| 13232 | 0,6402053 | 24343 | 0,4137731 | 35454 | -0,0340335 | 52115 | -0,06713416 |
| 13233 | 0,6206261 | 24344 | 0,2972409 | 35455 | -0,1549909 | 52121 | 0,21964523 |
| 13234 | 0,5040939 | 24345 | 0,1762834 | 35511 | 0,3374866 | 52122 | 0,14445769 |
| 13235 | 0,3831364 | 24351 | 0,3768766 | 35512 | 0,2622991 | 52123 | 0,12487853 |
| 13241 | 0,627801 | 24352 | 0,3016891 | 35513 | 0,2427199 | 52124 | 0,00834632 |
| 13242 | 0,5526135 | 24353 | 0,2821099 | 35514 | 0,1261877 | 52125 | -0,11261117 |
| 13243 | 0,5330343 | 24354 | 0,1655777 | 35515 | 0,0052302 | 52131 | 0,20835624 |
| 13244 | 0,4165021 | 24355 | 0,0446202 | 35521 | 0,2920096 | 52132 | 0,13316869 |
| 13245 | 0,2955446 | 24411 | 0,5962656 | 35522 | 0,2168221 | 52133 | 0,11358954 |
| 13251 | 0,4961378 | 24412 | 0,521078 | 35523 | 0,1972429 | 52134 | -0,00294268 |
| 13252 | 0,4209503 | 24413 | 0,5014989 | 35524 | 0,0807107 | 52135 | -0,12390016 |
| 13253 | 0,4013711 | 24414 | 0,3849667 | 35525 | -0,0402468 | 52141 | 0,12076442 |
| 13254 | 0,2848389 | 24415 | 0,2640092 | 35531 | 0,2807206 | 52142 | 0,04557687 |
| 13255 | 0,1638814 | 24421 | 0,5507886 | 35532 | 0,2055331 | 52143 | 0,02599772 |
| 13311 | 0,7566959 | 24422 | 0,475601 | 35533 | 0,1859539 | 52144 | -0,0905345 |
| 13312 | 0,6815084 | 24423 | 0,4560219 | 35534 | 0,0694217 | 52145 | -0,21149198 |
| 13313 | 0,6619292 | 24424 | 0,3394897 | 35535 | -0,0515358 | 52151 | -0,01089876 |
| 13314 | 0,545397 | 24425 | 0,2185322 | 35541 | 0,1931288 | 52152 | -0,08608631 |
| 13315 | 0,4244395 | 24431 | 0,5394996 | 35542 | 0,1179412 | 52153 | -0,10566546 |
| 13321 | 0,7112189 | 24432 | 0,464312 | 35543 | 0,0983621 | 52154 | -0,22219768 |
| 13322 | 0,6360314 | 24433 | 0,4447329 | 35544 | -0,0181701 | 52155 | -0,34315516 |
| 13323 | 0,6164522 | 24434 | 0,3282007 | 35545 | -0,1391276 | 52211 | 0,20241668 |
| 13324 | 0,49992 | 24435 | 0,2072432 | 35551 | 0,0614656 | 52212 | 0,12722914 |
| 13325 | 0,3789625 | 24441 | 0,4519078 | 35552 | -0,0137219 | 52213 | 0,10764998 |
| 13331 | 0,6999299 | 24442 | 0,3767202 | 35553 | -0,0333011 | 52214 | -0,00888223 |
| 13332 | 0,6247424 | 24443 | 0,3571411 | 35554 | -0,1498333 | 52215 | -0,12983972 |
| 13333 | 0,6051632 | 24444 | 0,2406088 | 35555 | -0,2707908 | 52221 | 0,15693968 |
| 13334 | 0,488631 | 24445 | 0,1196514 | 41111 | 0,8867717 | 52222 | 0,08175214 |
| 13335 | 0,3676735 | 24451 | 0,3202446 | 41112 | 0,8115841 | 52223 | 0,06217298 |
| 13341 | 0,6123381 | 24452 | 0,245057 | 41113 | 0,792005 | 52224 | -0,05435924 |
| 13342 | 0,5371505 | 24453 | 0,2254779 | 41114 | 0,6754727 | 52225 | -0,17531672 |
| 13343 | 0,5175714 | 24454 | 0,1089457 | 41115 | 0,5545153 | 52231 | 0,14565068 |
| 13344 | 0,4010392 | 24455 | -0,012012 | 41121 | 0,8412947 | 52232 | 0,07046314 |
| 13345 | 0,2800817 | 24511 | 0,4804657 | 41122 | 0,7661071 | 52233 | 0,05088398 |
| 13351 | 0,4806749 | 24512 | 0,4052782 | 41123 | 0,746528 | 52234 | -0,06564823 |
| 13352 | 0,4054874 | 24513 | 0,385699 | 41124 | 0,6299957 | 52235 | -0,18660572 |
| 13353 | 0,3859082 | 24514 | 0,2691668 | 41125 | 0,5090383 | 52241 | 0,05805886 |
| 13354 | 0,269376 | 24515 | 0,1482093 | 41131 | 0,8300057 | 52242 | -0,01712868 |
| 13355 | 0,1484185 | 24521 | 0,4349887 | 41132 | 0,7548181 | 52243 | -0,03670784 |
| 13411 | 0,7000639 | 24522 | 0,3598012 | 41133 | 0,735239 | 52244 | -0,15324005 |
| 13412 | 0,6248763 | 24523 | 0,340222 | 41134 | 0,6187067 | 52245 | -0,27419754 |
| 13413 | 0,6052972 | 24524 | 0,2236898 | 41135 | 0,4977493 | 52251 | -0,07360432 |
| 13414 | 0,4887649 | 24525 | 0,1027323 | 41141 | 0,7424138 | 52252 | -0,14879186 |
| 13415 | 0,3678075 | 24531 | 0,4236997 | 41142 | 0,6672263 | 52253 | -0,16837102 |
| 13421 | 0,6545869 | 24532 | 0,3485122 | 41143 | 0,6476471 | 52254 | -0,28490323 |
| 13422 | 0,5793993 | 24533 | 0,328933 | 41144 | 0,5311149 | 52255 | -0,40586072 |
| 13423 | 0,5598202 | 24534 | 0,2124008 | 41145 | 0,4101574 | 52311 | 0,18695374 |
| 13424 | 0,4432879 | 24535 | 0,0914433 | 41151 | 0,6107507 | 52312 | 0,11176619 |
| 13425 | 0,3223305 | 24541 | 0,3361079 | 41152 | 0,5355631 | 52313 | 0,09218704 |
| 13431 | 0,6432979 | 24542 | 0,2609204 | 41153 | 0,515984 | 52314 | -0,02434518 |
| 13432 | 0,5681103 | 24543 | 0,2413412 | 41154 | 0,3994517 | 52315 | -0,14530266 |
| 13433 | 0,5485312 | 24544 | 0,124809 | 41155 | 0,2784943 | 52321 | 0,14147673 |
| 13434 | 0,4319989 | 24545 | 0,0038515 | 41211 | 0,8240661 | 52322 | 0,06628919 |
| 13435 | 0,3110415 | 24551 | 0,2044447 | 41212 | 0,7488786 | 52323 | 0,04671003 |
| 13441 | 0,555706 | 24552 | 0,1292572 | 41213 | 0,7292994 | 52324 | -0,06982218 |
| 13442 | 0,4805185 | 24553 | 0,109678 | 41214 | 0,6127672 | 52325 | -0,19077967 |
| 13443 | 0,4609393 | 24554 | -0,006854 | 41215 | 0,4918097 | 52331 | 0,13018774 |
| 13444 | 0,3444071 | 24555 | -0,127812 | 41221 | 0,7785891 | 52332 | 0,05500019 |
| 13445 | 0,2234496 | 25111 | 0,6014945 | 41222 | 0,7034016 | 52333 | 0,03542104 |
| 13451 | 0,4240429 | 25112 | 0,5263069 | 41223 | 0,6838224 | 52334 | -0,08111118 |
| 13452 | 0,3488553 | 25113 | 0,5067278 | 41224 | 0,5672902 | 52335 | -0,20206866 |
| 13453 | 0,3292762 | 25114 | 0,3901956 | 41225 | 0,4463327 | 52341 | 0,04259592 |
| 13454 | 0,2127439 | 25115 | 0,2692381 | 41231 | 0,7673001 | 52342 | -0,03259163 |
| 13455 | 0,0917865 | 25121 | 0,5560175 | 41232 | 0,6921126 | 52343 | -0,05217078 |
| 13511 | 0,584264 | 25122 | 0,4808299 | 41233 | 0,6725334 | 52344 | -0,168703 |
| 13512 | 0,5090765 | 25123 | 0,4612508 | 41234 | 0,5560012 | 52345 | -0,28966048 |
| 13513 | 0,4894973 | 25124 | 0,3447186 | 41235 | 0,4350437 | 52351 | -0,08906726 |
| 13514 | 0,3729651 | 25125 | 0,2237611 | 41241 | 0,6797083 | 52352 | -0,16425481 |
| 13515 | 0,2520076 | 25131 | 0,5447285 | 41242 | 0,6045207 | 52353 | -0,18383396 |
| 13521 | 0,538787 | 25132 | 0,4695409 | 41243 | 0,5849416 | 52354 | -0,30036618 |
| 13522 | 0,4635995 | 25133 | 0,4499618 | 41244 | 0,4684094 | 52355 | -0,42132366 |
| 13523 | 0,4440203 | 25134 | 0,3334296 | 41245 | 0,3474519 | 52411 | 0,13032169 |
| 13524 | 0,3274881 | 25135 | 0,2124721 | 41251 | 0,5480451 | 52412 | 0,05513415 |
| 13525 | 0,2065306 | 25141 | 0,4571367 | 41252 | 0,4728576 | 52413 | 0,03555499 |
| 13531 | 0,527498 | 25142 | 0,3819491 | 41253 | 0,4532784 | 52414 | -0,08097722 |
| 13532 | 0,4523105 | 25143 | 0,36237 | 41254 | 0,3367462 | 52415 | -0,20193471 |
| 13533 | 0,4327313 | 25144 | 0,2458378 | 41255 | 0,2157887 | 52421 | 0,08484469 |
| 13534 | 0,3161991 | 25145 | 0,1248803 | 41311 | 0,8086032 | 52422 | 0,00965715 |
| 13535 | 0,1952416 | 25151 | 0,3254735 | 41312 | 0,7334156 | 52423 | -0,00992201 |
| 13541 | 0,4399062 | 25152 | 0,2502859 | 41313 | 0,7138365 | 52424 | -0,12645422 |
| 13542 | 0,3647186 | 25153 | 0,2307068 | 41314 | 0,5973042 | 52425 | -0,24741171 |
| 13543 | 0,3451395 | 25154 | 0,1141746 | 41315 | 0,4763468 | 52431 | 0,07355569 |
| 13544 | 0,2286073 | 25155 | -0,006783 | 41321 | 0,7631262 | 52432 | -0,00163185 |
| 13545 | 0,1076498 | 25211 | 0,5387889 | 41322 | 0,6879386 | 52433 | -0,02121101 |
| 13551 | 0,308243 | 25212 | 0,4636014 | 41323 | 0,6683595 | 52434 | -0,13774322 |
| 13552 | 0,2330555 | 25213 | 0,4440222 | 41324 | 0,5518272 | 52435 | -0,25870071 |
| 13553 | 0,2134763 | 25214 | 0,32749 | 41325 | 0,4308698 | 52441 | -0,01403613 |
| 13554 | 0,0969441 | 25215 | 0,2065325 | 41331 | 0,7518372 | 52442 | -0,08922367 |
| 13555 | -0,024013 | 25221 | 0,4933119 | 41332 | 0,6766496 | 52443 | -0,10880283 |
| 14111 | 0,7932588 | 25222 | 0,4181244 | 41333 | 0,6570705 | 52444 | -0,22533504 |
| 14112 | 0,7180713 | 25223 | 0,3985452 | 41334 | 0,5405382 | 52445 | -0,34629253 |
| 14113 | 0,6984921 | 25224 | 0,282013 | 41335 | 0,4195808 | 52451 | -0,14569931 |
| 14114 | 0,5819599 | 25225 | 0,1610555 | 41341 | 0,6642453 | 52452 | -0,22088685 |
| 14115 | 0,4610024 | 25231 | 0,4820229 | 41342 | 0,5890578 | 52453 | -0,24046601 |
| 14121 | 0,7477818 | 25232 | 0,4068354 | 41343 | 0,5694786 | 52454 | -0,35699822 |
| 14122 | 0,6725943 | 25233 | 0,3872562 | 41344 | 0,4529464 | 52455 | -0,47795571 |
| 14123 | 0,6530151 | 25234 | 0,270724 | 41345 | 0,3319889 | 52511 | 0,01452184 |
| 14124 | 0,5364829 | 25235 | 0,1497665 | 41351 | 0,5325822 | 52512 | -0,06066571 |
| 14125 | 0,4155254 | 25241 | 0,3944311 | 41352 | 0,4573946 | 52513 | -0,08024486 |
| 14131 | 0,7364928 | 25242 | 0,3192436 | 41353 | 0,4378155 | 52514 | -0,19677708 |
| 14132 | 0,6613053 | 25243 | 0,2996644 | 41354 | 0,3212832 | 52515 | -0,31773456 |
| 14133 | 0,6417261 | 25244 | 0,1831322 | 41355 | 0,2003258 | 52521 | -0,03095517 |
| 14134 | 0,5251939 | 25245 | 0,0621747 | 41411 | 0,7519711 | 52522 | -0,10614271 |
| 14135 | 0,4042364 | 25251 | 0,2627679 | 41412 | 0,6767836 | 52523 | -0,12572187 |
| 14141 | 0,648901 | 25252 | 0,1875804 | 41413 | 0,6572044 | 52524 | -0,24225408 |
| 14142 | 0,5737135 | 25253 | 0,1680012 | 41414 | 0,5406722 | 52525 | -0,36321157 |
| 14143 | 0,5541343 | 25254 | 0,051469 | 41415 | 0,4197147 | 52531 | -0,04224416 |
| 14144 | 0,4376021 | 25255 | -0,069488 | 41421 | 0,7064941 | 52532 | -0,11743171 |
| 14145 | 0,3166446 | 25311 | 0,523326 | 41422 | 0,6313066 | 52533 | -0,13701086 |
| 14151 | 0,5172378 | 25312 | 0,4481384 | 41423 | 0,6117274 | 52534 | -0,25354308 |
| 14152 | 0,4420503 | 25313 | 0,4285593 | 41424 | 0,4951952 | 52535 | -0,37450056 |
| 14153 | 0,4224711 | 25314 | 0,3120271 | 41425 | 0,3742377 | 52541 | -0,12983598 |
| 14154 | 0,3059389 | 25315 | 0,1910696 | 41431 | 0,6952051 | 52542 | -0,20502353 |
| 14155 | 0,1849814 | 25321 | 0,477849 | 41432 | 0,6200176 | 52543 | -0,22460268 |
| 14211 | 0,7305533 | 25322 | 0,4026614 | 41433 | 0,6004384 | 52544 | -0,3411349 |
| 14212 | 0,6553657 | 25323 | 0,3830823 | 41434 | 0,4839062 | 52545 | -0,46209238 |
| 14213 | 0,6357866 | 25324 | 0,2665501 | 41435 | 0,3629487 | 52551 | -0,26149916 |
| 14214 | 0,5192544 | 25325 | 0,1455926 | 41441 | 0,6076133 | 52552 | -0,33668671 |
| 14215 | 0,3982969 | 25331 | 0,46656 | 41442 | 0,5324257 | 52553 | -0,35626586 |
| 14221 | 0,6850763 | 25332 | 0,3913724 | 41443 | 0,5128466 | 52554 | -0,47279808 |
| 14222 | 0,6098887 | 25333 | 0,3717933 | 41444 | 0,3963144 | 52555 | -0,59375556 |
| 14223 | 0,5903096 | 25334 | 0,2552611 | 41445 | 0,2753569 | 53111 | 0,2374873 |
| 14224 | 0,4737774 | 25335 | 0,1343036 | 41451 | 0,4759501 | 53112 | 0,16229976 |
| 14225 | 0,3528199 | 25341 | 0,3789682 | 41452 | 0,4007626 | 53113 | 0,1427206 |
| 14231 | 0,6737873 | 25342 | 0,3037806 | 41453 | 0,3811834 | 53114 | 0,02618839 |
| 14232 | 0,5985997 | 25343 | 0,2842015 | 41454 | 0,2646512 | 53115 | -0,0947691 |
| 14233 | 0,5790206 | 25344 | 0,1676693 | 41455 | 0,1436937 | 53121 | 0,1920103 |
| 14234 | 0,4624884 | 25345 | 0,0467118 | 41511 | 0,6361713 | 53122 | 0,11682276 |
| 14235 | 0,3415309 | 25351 | 0,247305 | 41512 | 0,5609837 | 53123 | 0,0972436 |
| 14241 | 0,5861955 | 25352 | 0,1721174 | 41513 | 0,5414046 | 53124 | -0,01928862 |
| 14242 | 0,5110079 | 25353 | 0,1525383 | 41514 | 0,4248723 | 53125 | -0,1402461 |
| 14243 | 0,4914288 | 25354 | 0,0360061 | 41515 | 0,3039149 | 53131 | 0,1807213 |
| 14244 | 0,3748965 | 25355 | -0,084951 | 41521 | 0,5906943 | 53132 | 0,10553376 |
| 14245 | 0,2539391 | 25411 | 0,4666939 | 41522 | 0,5155067 | 53133 | 0,0859546 |
| 14251 | 0,4545323 | 25412 | 0,3915064 | 41523 | 0,4959276 | 53134 | -0,03057761 |
| 14252 | 0,3793447 | 25413 | 0,3719272 | 41524 | 0,3793953 | 53135 | -0,1515351 |
| 14253 | 0,3597656 | 25414 | 0,255395 | 41525 | 0,2584379 | 53141 | 0,09312948 |
| 14254 | 0,2432334 | 25415 | 0,1344375 | 41531 | 0,5794053 | 53142 | 0,01794194 |
| 14255 | 0,1222759 | 25421 | 0,4212169 | 41532 | 0,5042177 | 53143 | -0,00163722 |
| 14311 | 0,7150903 | 25422 | 0,3460294 | 41533 | 0,4846386 | 53144 | -0,11816943 |
| 14312 | 0,6399028 | 25423 | 0,3264502 | 41534 | 0,3681063 | 53145 | -0,23912692 |
| 14313 | 0,6203236 | 25424 | 0,209918 | 41535 | 0,2471489 | 53151 | -0,0385337 |
| 14314 | 0,5037914 | 25425 | 0,0889605 | 41541 | 0,4918134 | 53152 | -0,11372124 |
| 14315 | 0,3828339 | 25431 | 0,4099279 | 41542 | 0,4166259 | 53153 | -0,1333004 |
| 14321 | 0,6696133 | 25432 | 0,3347404 | 41543 | 0,3970467 | 53154 | -0,24983261 |
| 14322 | 0,5944258 | 25433 | 0,3151612 | 41544 | 0,2805145 | 53155 | -0,3707901 |
| 14323 | 0,5748466 | 25434 | 0,198629 | 41545 | 0,159557 | 53211 | 0,17478174 |
| 14324 | 0,4583144 | 25435 | 0,0776715 | 41551 | 0,3601503 | 53212 | 0,0995942 |
| 14325 | 0,3373569 | 25441 | 0,3223361 | 41552 | 0,2849627 | 53213 | 0,08001504 |
| 14331 | 0,6583243 | 25442 | 0,2471486 | 41553 | 0,2653836 | 53214 | -0,03651717 |
| 14332 | 0,5831368 | 25443 | 0,2275694 | 41554 | 0,1488513 | 53215 | -0,15747466 |
| 14333 | 0,5635576 | 25444 | 0,1110372 | 41555 | 0,0278939 | 53221 | 0,12930474 |
| 14334 | 0,4470254 | 25445 | -0,00992 | 42111 | 0,749271 | 53222 | 0,0541172 |
| 14335 | 0,3260679 | 25451 | 0,1906729 | 42112 | 0,6740835 | 53223 | 0,03453804 |
| 14341 | 0,5707325 | 25452 | 0,1154854 | 42113 | 0,6545043 | 53224 | -0,08199417 |
| 14342 | 0,495545 | 25453 | 0,0959062 | 42114 | 0,5379721 | 53225 | -0,20295166 |
| 14343 | 0,4759658 | 25454 | -0,020626 | 42115 | 0,4170146 | 53231 | 0,11801574 |
| 14344 | 0,3594336 | 25455 | -0,141583 | 42121 | 0,703794 | 53232 | 0,0428282 |
| 14345 | 0,2384761 | 25511 | 0,3508941 | 42122 | 0,6286065 | 53233 | 0,02324904 |
| 14351 | 0,4390693 | 25512 | 0,2757065 | 42123 | 0,6090273 | 53234 | -0,09328317 |
| 14352 | 0,3638818 | 25513 | 0,2561274 | 42124 | 0,4924951 | 53235 | -0,21424066 |
| 14353 | 0,3443026 | 25514 | 0,1395952 | 42125 | 0,3715376 | 53241 | 0,03042392 |
| 14354 | 0,2277704 | 25515 | 0,0186377 | 42131 | 0,692505 | 53242 | -0,04476362 |
| 14355 | 0,1068129 | 25521 | 0,3054171 | 42132 | 0,6173175 | 53243 | -0,06434278 |
| 14411 | 0,6584583 | 25522 | 0,2302295 | 42133 | 0,5977383 | 53244 | -0,18087499 |
| 14412 | 0,5832707 | 25523 | 0,2106504 | 42134 | 0,4812061 | 53245 | -0,30183248 |
| 14413 | 0,5636916 | 25524 | 0,0941182 | 42135 | 0,3602486 | 53251 | -0,10123926 |
| 14414 | 0,4471594 | 25525 | -0,026839 | 42141 | 0,6049132 | 53252 | -0,1764268 |
| 14415 | 0,3262019 | 25531 | 0,2941281 | 42142 | 0,5297256 | 53253 | -0,19600596 |
| 14421 | 0,6129813 | 25532 | 0,2189405 | 42143 | 0,5101465 | 53254 | -0,31253817 |
| 14422 | 0,5377937 | 25533 | 0,1993614 | 42144 | 0,3936143 | 53255 | -0,43349566 |
| 14423 | 0,5182146 | 25534 | 0,0828292 | 42145 | 0,2726568 | 53311 | 0,1593188 |
| 14424 | 0,4016824 | 25535 | -0,038128 | 42151 | 0,47325 | 53312 | 0,08413126 |
| 14425 | 0,2807249 | 25541 | 0,2065363 | 42152 | 0,3980625 | 53313 | 0,0645521 |
| 14431 | 0,6016923 | 25542 | 0,1313487 | 42153 | 0,3784833 | 53314 | -0,05198011 |
| 14432 | 0,5265047 | 25543 | 0,1117696 | 42154 | 0,2619511 | 53315 | -0,1729376 |
| 14433 | 0,5069256 | 25544 | -0,004763 | 42155 | 0,1409936 | 53321 | 0,1138418 |
| 14434 | 0,3903934 | 25545 | -0,12572 | 42211 | 0,6865654 | 53322 | 0,03865426 |
| 14435 | 0,2694359 | 25551 | 0,0748731 | 42212 | 0,6113779 | 53323 | 0,0190751 |
| 14441 | 0,5141005 | 25552 | -0,000314 | 42213 | 0,5917987 | 53324 | -0,09745712 |
| 14442 | 0,4389129 | 25553 | -0,019894 | 42214 | 0,4752665 | 53325 | -0,2184146 |
| 14443 | 0,4193338 | 25554 | -0,136426 | 42215 | 0,354309 | 53331 | 0,1025528 |
| 14444 | 0,3028016 | 25555 | -0,257383 | 42221 | 0,6410884 | 53332 | 0,02736526 |
| 14445 | 0,1818441 | 31111 | 0,9243998 | 42222 | 0,5659009 | 53333 | 0,0077861 |
| 14451 | 0,3824373 | 31112 | 0,8492123 | 42223 | 0,5463217 | 53334 | -0,10874611 |
| 14452 | 0,3072497 | 31113 | 0,8296331 | 42224 | 0,4297895 | 53335 | -0,2297036 |
| 14453 | 0,2876706 | 31114 | 0,7131009 | 42225 | 0,308832 | 53341 | 0,01496098 |
| 14454 | 0,1711384 | 31115 | 0,5921434 | 42231 | 0,6297994 | 53342 | -0,06022656 |
| 14455 | 0,0501809 | 31121 | 0,8789228 | 42232 | 0,5546119 | 53343 | -0,07980572 |
| 14511 | 0,5426584 | 31122 | 0,8037353 | 42233 | 0,5350327 | 53344 | -0,19633793 |
| 14512 | 0,4674709 | 31123 | 0,7841561 | 42234 | 0,4185005 | 53345 | -0,31729542 |
| 14513 | 0,4478917 | 31124 | 0,6676239 | 42235 | 0,297543 | 53351 | -0,1167022 |
| 14514 | 0,3313595 | 31125 | 0,5466664 | 42241 | 0,5422076 | 53352 | -0,19188974 |
| 14515 | 0,210402 | 31131 | 0,8676338 | 42242 | 0,4670201 | 53353 | -0,2114689 |
| 14521 | 0,4971814 | 31132 | 0,7924463 | 42243 | 0,4474409 | 53354 | -0,32800111 |
| 14522 | 0,4219939 | 31133 | 0,7728671 | 42244 | 0,3309087 | 53355 | -0,4489586 |
| 14523 | 0,4024147 | 31134 | 0,6563349 | 42245 | 0,2099512 | 53411 | 0,10268675 |
| 14524 | 0,2858825 | 31135 | 0,5353774 | 42251 | 0,4105444 | 53412 | 0,02749921 |
| 14525 | 0,164925 | 31141 | 0,780042 | 42252 | 0,3353569 | 53413 | 0,00792005 |
| 14531 | 0,4858924 | 31142 | 0,7048544 | 42253 | 0,3157777 | 53414 | -0,10861216 |
| 14532 | 0,4107049 | 31143 | 0,6852753 | 42254 | 0,1992455 | 53415 | -0,22956965 |
| 14533 | 0,3911257 | 31144 | 0,5687431 | 42255 | 0,078288 | 53421 | 0,05720975 |
| 14534 | 0,2745935 | 31145 | 0,4477856 | 42311 | 0,6711025 | 53422 | -0,01797779 |
| 14535 | 0,153636 | 31151 | 0,6483788 | 42312 | 0,595915 | 53423 | -0,03755695 |
| 14541 | 0,3983006 | 31152 | 0,5731913 | 42313 | 0,5763358 | 53424 | -0,15408916 |
| 14542 | 0,3231131 | 31153 | 0,5536121 | 42314 | 0,4598036 | 53425 | -0,27504665 |
| 14543 | 0,3035339 | 31154 | 0,4370799 | 42315 | 0,3388461 | 53431 | 0,04592075 |
| 14544 | 0,1870017 | 31155 | 0,3161224 | 42321 | 0,6256255 | 53432 | -0,02926679 |
| 14545 | 0,0660442 | 31211 | 0,8616942 | 42322 | 0,550438 | 53433 | -0,04884595 |
| 14551 | 0,2666374 | 31212 | 0,7865067 | 42323 | 0,5308588 | 53434 | -0,16537816 |
| 14552 | 0,1914499 | 31213 | 0,7669275 | 42324 | 0,4143266 | 53435 | -0,28633565 |
| 14553 | 0,1718707 | 31214 | 0,6503953 | 42325 | 0,2933691 | 53441 | -0,04167107 |
| 14554 | 0,0553385 | 31215 | 0,5294378 | 42331 | 0,6143365 | 53442 | -0,11685861 |
| 14555 | -0,065619 | 31221 | 0,8162172 | 42332 | 0,539149 | 53443 | -0,13643777 |
| 15111 | 0,6636872 | 31222 | 0,7410297 | 42333 | 0,5195698 | 53444 | -0,25296998 |
| 15112 | 0,5884997 | 31223 | 0,7214505 | 42334 | 0,4030376 | 53445 | -0,37392747 |
| 15113 | 0,5689205 | 31224 | 0,6049183 | 42335 | 0,2820801 | 53451 | -0,17333425 |
| 15114 | 0,4523883 | 31225 | 0,4839608 | 42341 | 0,5267447 | 53452 | -0,24852179 |
| 15115 | 0,3314308 | 31231 | 0,8049282 | 42342 | 0,4515571 | 53453 | -0,26810095 |
| 15121 | 0,6182102 | 31232 | 0,7297407 | 42343 | 0,431978 | 53454 | -0,38463316 |
| 15122 | 0,5430227 | 31233 | 0,7101615 | 42344 | 0,3154458 | 53455 | -0,50559065 |
| 15123 | 0,5234435 | 31234 | 0,5936293 | 42345 | 0,1944883 | 53511 | -0,0131131 |
| 15124 | 0,4069113 | 31235 | 0,4726718 | 42351 | 0,3950815 | 53512 | -0,08830064 |
| 15125 | 0,2859538 | 31241 | 0,7173364 | 42352 | 0,319894 | 53513 | -0,1078798 |
| 15131 | 0,6069212 | 31242 | 0,6421489 | 42353 | 0,3003148 | 53514 | -0,22441201 |
| 15132 | 0,5317337 | 31243 | 0,6225697 | 42354 | 0,1837826 | 53515 | -0,3453695 |
| 15133 | 0,5121545 | 31244 | 0,5060375 | 42355 | 0,0628251 | 53521 | -0,0585901 |
| 15134 | 0,3956223 | 31245 | 0,38508 | 42411 | 0,6144704 | 53522 | -0,13377764 |
| 15135 | 0,2746648 | 31251 | 0,5856732 | 42412 | 0,5392829 | 53523 | -0,1533568 |
| 15141 | 0,5193294 | 31252 | 0,5104857 | 42413 | 0,5197037 | 53524 | -0,26988902 |
| 15142 | 0,4441418 | 31253 | 0,4909065 | 42414 | 0,4031715 | 53525 | -0,3908465 |
| 15143 | 0,4245627 | 31254 | 0,3743743 | 42415 | 0,282214 | 53531 | -0,0698791 |
| 15144 | 0,3080305 | 31255 | 0,2534168 | 42421 | 0,5689934 | 53532 | -0,14506664 |
| 15145 | 0,187073 | 31311 | 0,8462313 | 42422 | 0,4938059 | 53533 | -0,1646458 |
| 15151 | 0,3876662 | 31312 | 0,7710438 | 42423 | 0,4742267 | 53534 | -0,28117801 |
| 15152 | 0,3124787 | 31313 | 0,7514646 | 42424 | 0,3576945 | 53535 | -0,4021355 |
| 15153 | 0,2928995 | 31314 | 0,6349324 | 42425 | 0,236737 | 53541 | -0,15747092 |
| 15154 | 0,1763673 | 31315 | 0,5139749 | 42431 | 0,5577044 | 53542 | -0,23265846 |
| 15155 | 0,0554098 | 31321 | 0,8007543 | 42432 | 0,4825169 | 53543 | -0,25223762 |
| 15211 | 0,6009816 | 31322 | 0,7255668 | 42433 | 0,4629377 | 53544 | -0,36876983 |
| 15212 | 0,5257941 | 31323 | 0,7059876 | 42434 | 0,3464055 | 53545 | -0,48972732 |
| 15213 | 0,5062149 | 31324 | 0,5894554 | 42435 | 0,225448 | 53551 | -0,2891341 |
| 15214 | 0,3896827 | 31325 | 0,4684979 | 42441 | 0,4701126 | 53552 | -0,36432164 |
| 15215 | 0,2687252 | 31331 | 0,7894653 | 42442 | 0,3949251 | 53553 | -0,3839008 |
| 15221 | 0,5555046 | 31332 | 0,7142778 | 42443 | 0,3753459 | 53554 | -0,50043301 |
| 15222 | 0,4803171 | 31333 | 0,6946986 | 42444 | 0,2588137 | 53555 | -0,6213905 |
| 15223 | 0,4607379 | 31334 | 0,5781664 | 42445 | 0,1378562 | 54111 | 0,19588173 |
| 15224 | 0,3442057 | 31335 | 0,4572089 | 42451 | 0,3384494 | 54112 | 0,12069419 |
| 15225 | 0,2232482 | 31341 | 0,7018735 | 42452 | 0,2632619 | 54113 | 0,10111503 |
| 15231 | 0,5442156 | 31342 | 0,6266859 | 42453 | 0,2436827 | 54114 | -0,01541718 |
| 15232 | 0,4690281 | 31343 | 0,6071068 | 42454 | 0,1271505 | 54115 | -0,13637467 |
| 15233 | 0,4494489 | 31344 | 0,4905746 | 42455 | 0,006193 | 54121 | 0,15040473 |
| 15234 | 0,3329167 | 31345 | 0,3696171 | 42511 | 0,4986706 | 54122 | 0,07521719 |
| 15235 | 0,2119592 | 31351 | 0,5702103 | 42512 | 0,4234831 | 54123 | 0,05563803 |
| 15241 | 0,4566238 | 31352 | 0,4950228 | 42513 | 0,4039039 | 54124 | -0,06089419 |
| 15242 | 0,3814363 | 31353 | 0,4754436 | 42514 | 0,2873717 | 54125 | -0,18185167 |
| 15243 | 0,3618571 | 31354 | 0,3589114 | 42515 | 0,1664142 | 54131 | 0,13911573 |
| 15244 | 0,2453249 | 31355 | 0,2379539 | 42521 | 0,4531936 | 54132 | 0,06392819 |
| 15245 | 0,1243674 | 31411 | 0,7895993 | 42522 | 0,3780061 | 54133 | 0,04434903 |
| 15251 | 0,3249606 | 31412 | 0,7144117 | 42523 | 0,3584269 | 54134 | -0,07218318 |
| 15252 | 0,2497731 | 31413 | 0,6948326 | 42524 | 0,2418947 | 54135 | -0,19314067 |
| 15253 | 0,2301939 | 31414 | 0,5783003 | 42525 | 0,1209372 | 54141 | 0,05152391 |
| 15254 | 0,1136617 | 31415 | 0,4573429 | 42531 | 0,4419046 | 54142 | -0,02366363 |
| 15255 | -0,007296 | 31421 | 0,7441223 | 42532 | 0,3667171 | 54143 | -0,04324279 |
| 15311 | 0,5855187 | 31422 | 0,6689347 | 42533 | 0,3471379 | 54144 | -0,159775 |
| 15312 | 0,5103312 | 31423 | 0,6493556 | 42534 | 0,2306057 | 54145 | -0,28073249 |
| 15313 | 0,490752 | 31424 | 0,5328233 | 42535 | 0,1096482 | 54151 | -0,08013927 |
| 15314 | 0,3742198 | 31425 | 0,4118659 | 42541 | 0,3543128 | 54152 | -0,15532681 |
| 15315 | 0,2532623 | 31431 | 0,7328333 | 42542 | 0,2791252 | 54153 | -0,17490597 |
| 15321 | 0,5400417 | 31432 | 0,6576457 | 42543 | 0,2595461 | 54154 | -0,29143818 |
| 15322 | 0,4648542 | 31433 | 0,6380666 | 42544 | 0,1430139 | 54155 | -0,41239567 |
| 15323 | 0,445275 | 31434 | 0,5215343 | 42545 | 0,0220564 | 54211 | 0,13317617 |
| 15324 | 0,3287428 | 31435 | 0,4005769 | 42551 | 0,2226496 | 54212 | 0,05798863 |
| 15325 | 0,2077853 | 31441 | 0,6452414 | 42552 | 0,1474621 | 54213 | 0,03840947 |
| 15331 | 0,5287527 | 31442 | 0,5700539 | 42553 | 0,1278829 | 54214 | -0,07812274 |
| 15332 | 0,4535652 | 31443 | 0,5504747 | 42554 | 0,0113507 | 54215 | -0,19908023 |
| 15333 | 0,433986 | 31444 | 0,4339425 | 42555 | -0,1096068 | 54221 | 0,08769917 |
| 15334 | 0,3174538 | 31445 | 0,312985 | 43111 | 0,7216361 | 54222 | 0,01251163 |
| 15335 | 0,1964963 | 31451 | 0,5135783 | 43112 | 0,6464485 | 54223 | -0,00706753 |
| 15341 | 0,4411609 | 31452 | 0,4383907 | 43113 | 0,6268694 | 54224 | -0,12359974 |
| 15342 | 0,3659733 | 31453 | 0,4188116 | 43114 | 0,5103371 | 54225 | -0,24455723 |
| 15343 | 0,3463942 | 31454 | 0,3022793 | 43115 | 0,3893797 | 54231 | 0,07641017 |
| 15344 | 0,229862 | 31455 | 0,1813219 | 43121 | 0,6761591 | 54232 | 0,00122263 |
| 15345 | 0,1089045 | 31511 | 0,6737994 | 43122 | 0,6009715 | 54233 | -0,01835653 |
| 15351 | 0,3094977 | 31512 | 0,5986119 | 43123 | 0,5813924 | 54234 | -0,13488874 |
| 15352 | 0,2343102 | 31513 | 0,5790327 | 43124 | 0,4648601 | 54235 | -0,25584623 |
| 15353 | 0,214731 | 31514 | 0,4625005 | 43125 | 0,3439027 | 54241 | -0,01118165 |
| 15354 | 0,0981988 | 31515 | 0,341543 | 43131 | 0,6648701 | 54242 | -0,08636919 |
| 15355 | -0,022759 | 31521 | 0,6283224 | 43132 | 0,5896825 | 54243 | -0,10594835 |
| 15411 | 0,5288867 | 31522 | 0,5531349 | 43133 | 0,5701034 | 54244 | -0,22248056 |
| 15412 | 0,4536991 | 31523 | 0,5335557 | 43134 | 0,4535711 | 54245 | -0,34343805 |
| 15413 | 0,43412 | 31524 | 0,4170235 | 43135 | 0,3326137 | 54251 | -0,14284483 |
| 15414 | 0,3175877 | 31525 | 0,296066 | 43141 | 0,5772782 | 54252 | -0,21803237 |
| 15415 | 0,1966303 | 31531 | 0,6170334 | 43142 | 0,5020907 | 54253 | -0,23761153 |
| 15421 | 0,4834097 | 31532 | 0,5418459 | 43143 | 0,4825115 | 54254 | -0,35414374 |
| 15422 | 0,4082221 | 31533 | 0,5222667 | 43144 | 0,3659793 | 54255 | -0,47510123 |
| 15423 | 0,388643 | 31534 | 0,4057345 | 43145 | 0,2450218 | 54311 | 0,11771323 |
| 15424 | 0,2721107 | 31535 | 0,284777 | 43151 | 0,4456151 | 54312 | 0,04252569 |
| 15425 | 0,1511533 | 31541 | 0,5294416 | 43152 | 0,3704275 | 54313 | 0,02294653 |
| 15431 | 0,4721207 | 31542 | 0,454254 | 43153 | 0,3508484 | 54314 | -0,09358568 |
| 15432 | 0,3969331 | 31543 | 0,4346749 | 43154 | 0,2343161 | 54315 | -0,21454317 |
| 15433 | 0,377354 | 31544 | 0,3181427 | 43155 | 0,1133587 | 54321 | 0,07223623 |
| 15434 | 0,2608217 | 31545 | 0,1971852 | 43211 | 0,6589305 | 54322 | -0,00295131 |
| 15435 | 0,1398643 | 31551 | 0,3977784 | 43212 | 0,583743 | 54323 | -0,02253047 |
| 15441 | 0,3845288 | 31552 | 0,3225909 | 43213 | 0,5641638 | 54324 | -0,13906269 |
| 15442 | 0,3093413 | 31553 | 0,3030117 | 43214 | 0,4476316 | 54325 | -0,26002017 |
| 15443 | 0,2897621 | 31554 | 0,1864795 | 43215 | 0,3266741 | 54331 | 0,06094723 |
| 15444 | 0,1732299 | 31555 | 0,065522 | 43221 | 0,6134535 | 54332 | -0,01424031 |
| 15445 | 0,0522724 | 32111 | 0,7868991 | 43222 | 0,538266 | 54333 | -0,03381947 |
| 15451 | 0,2528657 | 32112 | 0,7117116 | 43223 | 0,5186868 | 54334 | -0,15035168 |
| 15452 | 0,1776781 | 32113 | 0,6921324 | 43224 | 0,4021546 | 54335 | -0,27130917 |
| 15453 | 0,158099 | 32114 | 0,5756002 | 43225 | 0,2811971 | 54341 | -0,02664459 |
| 15454 | 0,0415667 | 32115 | 0,4546427 | 43231 | 0,6021645 | 54342 | -0,10183213 |
| 15455 | -0,079391 | 32121 | 0,7414221 | 43232 | 0,526977 | 54343 | -0,12141129 |
| 15511 | 0,4130868 | 32122 | 0,6662346 | 43233 | 0,5073978 | 54344 | -0,2379435 |
| 15512 | 0,3378993 | 32123 | 0,6466554 | 43234 | 0,3908656 | 54345 | -0,35890099 |
| 15513 | 0,3183201 | 32124 | 0,5301232 | 43235 | 0,2699081 | 54351 | -0,15830777 |
| 15514 | 0,2017879 | 32125 | 0,4091657 | 43241 | 0,5145727 | 54352 | -0,23349531 |
| 15515 | 0,0808304 | 32131 | 0,7301331 | 43242 | 0,4393851 | 54353 | -0,25307447 |
| 15521 | 0,3676098 | 32132 | 0,6549456 | 43243 | 0,419806 | 54354 | -0,36960668 |
| 15522 | 0,2924223 | 32133 | 0,6353664 | 43244 | 0,3032738 | 54355 | -0,49056417 |
| 15523 | 0,2728431 | 32134 | 0,5188342 | 43245 | 0,1823163 | 54411 | 0,06108118 |
| 15524 | 0,1563109 | 32135 | 0,3978767 | 43251 | 0,3829095 | 54412 | -0,01410636 |
| 15525 | 0,0353534 | 32141 | 0,6425413 | 43252 | 0,307722 | 54413 | -0,03368552 |
| 15531 | 0,3563208 | 32142 | 0,5673538 | 43253 | 0,2881428 | 54414 | -0,15021773 |
| 15532 | 0,2811333 | 32143 | 0,5477746 | 43254 | 0,1716106 | 54415 | -0,27117522 |
| 15533 | 0,2615541 | 32144 | 0,4312424 | 43255 | 0,0506531 | 54421 | 0,01560418 |
| 15534 | 0,1450219 | 32145 | 0,3102849 | 43311 | 0,6434676 | 54422 | -0,05958336 |
| 15535 | 0,0240644 | 32151 | 0,5108781 | 43312 | 0,56828 | 54423 | -0,07916252 |
| 15541 | 0,268729 | 32152 | 0,4356906 | 43313 | 0,5487009 | 54424 | -0,19569473 |
| 15542 | 0,1935414 | 32153 | 0,4161114 | 43314 | 0,4321686 | 54425 | -0,31665222 |
| 15543 | 0,1739623 | 32154 | 0,2995792 | 43315 | 0,3112112 | 54431 | 0,00431518 |
| 15544 | 0,0574301 | 32155 | 0,1786217 | 43321 | 0,5979906 | 54432 | -0,07087236 |
| 15545 | -0,063527 | 32211 | 0,7241936 | 43322 | 0,522803 | 54433 | -0,09045152 |
| 15551 | 0,1370658 | 32212 | 0,649006 | 43323 | 0,5032239 | 54434 | -0,20698373 |
| 15552 | 0,0618783 | 32213 | 0,6294269 | 43324 | 0,3866916 | 54435 | -0,32794122 |
| 15553 | 0,0422991 | 32214 | 0,5128947 | 43325 | 0,2657342 | 54441 | -0,08327664 |
| 15554 | -0,074233 | 32215 | 0,3919372 | 43331 | 0,5867016 | 54442 | -0,15846418 |
| 15555 | -0,195191 | 32221 | 0,6787166 | 43332 | 0,511514 | 54443 | -0,17804334 |
| 21111 | 0,9378073 | 32222 | 0,603529 | 43333 | 0,4919349 | 54444 | -0,29457555 |
| 21112 | 0,8626197 | 32223 | 0,5839499 | 43334 | 0,3754026 | 54445 | -0,41553304 |
| 21113 | 0,8430406 | 32224 | 0,4674177 | 43335 | 0,2544452 | 54451 | -0,21493982 |
| 21114 | 0,7265084 | 32225 | 0,3464602 | 43341 | 0,4991097 | 54452 | -0,29012736 |
| 21115 | 0,6055509 | 32231 | 0,6674276 | 43342 | 0,4239222 | 54453 | -0,30970652 |
| 21121 | 0,8923303 | 32232 | 0,59224 | 43343 | 0,404343 | 54454 | -0,42623873 |
| 21122 | 0,8171427 | 32233 | 0,5726609 | 43344 | 0,2878108 | 54455 | -0,54719622 |
| 21123 | 0,7975636 | 32234 | 0,4561287 | 43345 | 0,1668533 | 54511 | -0,05471867 |
| 21124 | 0,6810314 | 32235 | 0,3351712 | 43351 | 0,3674466 | 54512 | -0,12990621 |
| 21125 | 0,5600739 | 32241 | 0,5798358 | 43352 | 0,292259 | 54513 | -0,14948537 |
| 21131 | 0,8810413 | 32242 | 0,5046482 | 43353 | 0,2726799 | 54514 | -0,26601758 |
| 21132 | 0,8058537 | 32243 | 0,4850691 | 43354 | 0,1561476 | 54515 | -0,38697507 |
| 21133 | 0,7862746 | 32244 | 0,3685368 | 43355 | 0,0351902 | 54521 | -0,10019567 |
| 21134 | 0,6697424 | 32245 | 0,2475794 | 43411 | 0,5868355 | 54522 | -0,17538321 |
| 21135 | 0,5487849 | 32251 | 0,4481726 | 43412 | 0,511648 | 54523 | -0,19496237 |
| 21141 | 0,7934495 | 32252 | 0,372985 | 43413 | 0,4920688 | 54524 | -0,31149459 |
| 21142 | 0,7182619 | 32253 | 0,3534059 | 43414 | 0,3755366 | 54525 | -0,43245207 |
| 21143 | 0,6986828 | 32254 | 0,2368737 | 43415 | 0,2545791 | 54531 | -0,11148467 |
| 21144 | 0,5821506 | 32255 | 0,1159162 | 43421 | 0,5413585 | 54532 | -0,18667221 |
| 21145 | 0,4611931 | 32311 | 0,7087306 | 43422 | 0,466171 | 54533 | -0,20625137 |
| 21151 | 0,6617863 | 32312 | 0,6335431 | 43423 | 0,4465918 | 54534 | -0,32278358 |
| 21152 | 0,5865987 | 32313 | 0,6139639 | 43424 | 0,3300596 | 54535 | -0,44374107 |
| 21153 | 0,5670196 | 32314 | 0,4974317 | 43425 | 0,2091021 | 54541 | -0,19907649 |
| 21154 | 0,4504874 | 32315 | 0,3764742 | 43431 | 0,5300695 | 54542 | -0,27426403 |
| 21155 | 0,3295299 | 32321 | 0,6632536 | 43432 | 0,454882 | 54543 | -0,29384319 |
| 21211 | 0,8751017 | 32322 | 0,5880661 | 43433 | 0,4353028 | 54544 | -0,4103754 |
| 21212 | 0,7999142 | 32323 | 0,5684869 | 43434 | 0,3187706 | 54545 | -0,53133289 |
| 21213 | 0,780335 | 32324 | 0,4519547 | 43435 | 0,1978131 | 54551 | -0,33073967 |
| 21214 | 0,6638028 | 32325 | 0,3309972 | 43441 | 0,4424777 | 54552 | -0,40592721 |
| 21215 | 0,5428453 | 32331 | 0,6519646 | 43442 | 0,3672901 | 54553 | -0,42550637 |
| 21221 | 0,8296247 | 32332 | 0,5767771 | 43443 | 0,347711 | 54554 | -0,54203858 |
| 21222 | 0,7544372 | 32333 | 0,5571979 | 43444 | 0,2311788 | 54555 | -0,66299607 |
| 21223 | 0,734858 | 32334 | 0,4406657 | 43445 | 0,1102213 | 55111 | 0,0663101 |
| 21224 | 0,6183258 | 32335 | 0,3197082 | 43451 | 0,3108145 | 55112 | -0,00887744 |
| 21225 | 0,4973683 | 32341 | 0,5643728 | 43452 | 0,235627 | 55113 | -0,0284566 |
| 21231 | 0,8183357 | 32342 | 0,4891853 | 43453 | 0,2160478 | 55114 | -0,14498881 |
| 21232 | 0,7431482 | 32343 | 0,4696061 | 43454 | 0,0995156 | 55115 | -0,2659463 |
| 21233 | 0,723569 | 32344 | 0,3530739 | 43455 | -0,0214419 | 55121 | 0,0208331 |
| 21234 | 0,6070368 | 32345 | 0,2321164 | 43511 | 0,4710357 | 55122 | -0,05435444 |
| 21235 | 0,4860793 | 32351 | 0,4327096 | 43512 | 0,3958481 | 55123 | -0,0739336 |
| 21241 | 0,7307439 | 32352 | 0,3575221 | 43513 | 0,376269 | 55124 | -0,19046582 |
| 21242 | 0,6555564 | 32353 | 0,3379429 | 43514 | 0,2597367 | 55125 | -0,3114233 |
| 21243 | 0,6359772 | 32354 | 0,2214107 | 43515 | 0,1387793 | 55131 | 0,0095441 |
| 21244 | 0,519445 | 32355 | 0,1004532 | 43521 | 0,4255587 | 55132 | -0,06564344 |
| 21245 | 0,3984875 | 32411 | 0,6520986 | 43522 | 0,3503711 | 55133 | -0,0852226 |
| 21251 | 0,5990807 | 32412 | 0,576911 | 43523 | 0,330792 | 55134 | -0,20175481 |
| 21252 | 0,5238932 | 32413 | 0,5573319 | 43524 | 0,2142597 | 55135 | -0,3227123 |
| 21253 | 0,504314 | 32414 | 0,4407997 | 43525 | 0,0933023 | 55141 | -0,07804772 |
| 21254 | 0,3877818 | 32415 | 0,3198422 | 43531 | 0,4142697 | 55142 | -0,15323526 |
| 21255 | 0,2668243 | 32421 | 0,6066216 | 43532 | 0,3390821 | 55143 | -0,17281442 |
| 21311 | 0,8596388 | 32422 | 0,531434 | 43533 | 0,319503 | 55144 | -0,28934663 |
| 21312 | 0,7844512 | 32423 | 0,5118549 | 43534 | 0,2029707 | 55145 | -0,41030412 |
| 21313 | 0,7648721 | 32424 | 0,3953227 | 43535 | 0,0820133 | 55151 | -0,2097109 |
| 21314 | 0,6483399 | 32425 | 0,2743652 | 43541 | 0,3266778 | 55152 | -0,28489844 |
| 21315 | 0,5273824 | 32431 | 0,5953326 | 43542 | 0,2514903 | 55153 | -0,3044776 |
| 21321 | 0,8141618 | 32432 | 0,520145 | 43543 | 0,2319111 | 55154 | -0,42100981 |
| 21322 | 0,7389742 | 32433 | 0,5005659 | 43544 | 0,1153789 | 55155 | -0,5419673 |
| 21323 | 0,7193951 | 32434 | 0,3840337 | 43545 | -0,0055786 | 55211 | 0,00360454 |
| 21324 | 0,6028629 | 32435 | 0,2630762 | 43551 | 0,1950147 | 55212 | -0,071583 |
| 21325 | 0,4819054 | 32441 | 0,5077408 | 43552 | 0,1198271 | 55213 | -0,09116216 |
| 21331 | 0,8028728 | 32442 | 0,4325532 | 43553 | 0,100248 | 55214 | -0,20769437 |
| 21332 | 0,7276852 | 32443 | 0,4129741 | 43554 | -0,0162843 | 55215 | -0,32865186 |
| 21333 | 0,7081061 | 32444 | 0,2964419 | 43555 | -0,1372417 | 55221 | -0,04187246 |
| 21334 | 0,5915739 | 32445 | 0,1754844 | 44111 | 0,6800305 | 55222 | -0,11706 |
| 21335 | 0,4706164 | 32451 | 0,3760776 | 44112 | 0,6048429 | 55223 | -0,13663916 |
| 21341 | 0,715281 | 32452 | 0,30089 | 44113 | 0,5852638 | 55224 | -0,25317137 |
| 21342 | 0,6400934 | 32453 | 0,2813109 | 44114 | 0,4687316 | 55225 | -0,37412886 |
| 21343 | 0,6205143 | 32454 | 0,1647787 | 44115 | 0,3477741 | 55231 | -0,05316146 |
| 21344 | 0,5039821 | 32455 | 0,0438212 | 44121 | 0,6345535 | 55232 | -0,128349 |
| 21345 | 0,3830246 | 32511 | 0,5362987 | 44122 | 0,5593659 | 55233 | -0,14792816 |
| 21351 | 0,5836178 | 32512 | 0,4611112 | 44123 | 0,5397868 | 55234 | -0,26446037 |
| 21352 | 0,5084302 | 32513 | 0,441532 | 44124 | 0,4232546 | 55235 | -0,38541786 |
| 21353 | 0,4888511 | 32514 | 0,3249998 | 44125 | 0,3022971 | 55241 | -0,14075328 |
| 21354 | 0,3723189 | 32515 | 0,2040423 | 44131 | 0,6232645 | 55242 | -0,21594082 |
| 21355 | 0,2513614 | 32521 | 0,4908217 | 44132 | 0,5480769 | 55243 | -0,23551998 |
| 21411 | 0,8030067 | 32522 | 0,4156342 | 44133 | 0,5284978 | 55244 | -0,35205219 |
| 21412 | 0,7278192 | 32523 | 0,396055 | 44134 | 0,4119656 | 55245 | -0,47300968 |
| 21413 | 0,70824 | 32524 | 0,2795228 | 44135 | 0,2910081 | 55251 | -0,27241646 |
| 21414 | 0,5917078 | 32525 | 0,1585653 | 44141 | 0,5356727 | 55252 | -0,347604 |
| 21415 | 0,4707503 | 32531 | 0,4795327 | 44142 | 0,4604851 | 55253 | -0,36718316 |
| 21421 | 0,7575297 | 32532 | 0,4043452 | 44143 | 0,440906 | 55254 | -0,48371537 |
| 21422 | 0,6823422 | 32533 | 0,384766 | 44144 | 0,3243738 | 55255 | -0,60467286 |
| 21423 | 0,662763 | 32534 | 0,2682338 | 44145 | 0,2034163 | 55311 | -0,0118584 |
| 21424 | 0,5462308 | 32535 | 0,1472763 | 44151 | 0,4040095 | 55312 | -0,08704594 |
| 21425 | 0,4252733 | 32541 | 0,3919409 | 44152 | 0,3288219 | 55313 | -0,1066251 |
| 21431 | 0,7462407 | 32542 | 0,3167534 | 44153 | 0,3092428 | 55314 | -0,22315731 |
| 21432 | 0,6710532 | 32543 | 0,2971742 | 44154 | 0,1927106 | 55315 | -0,3441148 |
| 21433 | 0,651474 | 32544 | 0,180642 | 44155 | 0,0717531 | 55321 | -0,0573354 |
| 21434 | 0,5349418 | 32545 | 0,0596845 | 44211 | 0,6173249 | 55322 | -0,13252294 |
| 21435 | 0,4139843 | 32551 | 0,2602777 | 44212 | 0,5421374 | 55323 | -0,1521021 |
| 21441 | 0,6586489 | 32552 | 0,1850902 | 44213 | 0,5225582 | 55324 | -0,26863432 |
| 21442 | 0,5834614 | 32553 | 0,165511 | 44214 | 0,406026 | 55325 | -0,3895918 |
| 21443 | 0,5638822 | 32554 | 0,0489788 | 44215 | 0,2850685 | 55331 | -0,0686244 |
| 21444 | 0,44735 | 32555 | -0,071979 | 44221 | 0,5718479 | 55332 | -0,14381194 |
| 21445 | 0,3263925 | 33111 | 0,7592642 | 44222 | 0,4966604 | 55333 | -0,1633911 |
| 21451 | 0,5269857 | 33112 | 0,6840767 | 44223 | 0,4770812 | 55334 | -0,27992331 |
| 21452 | 0,4517982 | 33113 | 0,6644975 | 44224 | 0,360549 | 55335 | -0,4008808 |
| 21453 | 0,432219 | 33114 | 0,5479653 | 44225 | 0,2395915 | 55341 | -0,15621622 |
| 21454 | 0,3156868 | 33115 | 0,4270078 | 44231 | 0,5605589 | 55342 | -0,23140376 |
| 21455 | 0,1947293 | 33121 | 0,7137872 | 44232 | 0,4853714 | 55343 | -0,25098292 |
| 21511 | 0,6872069 | 33122 | 0,6385997 | 44233 | 0,4657922 | 55344 | -0,36751513 |
| 21512 | 0,6120193 | 33123 | 0,6190205 | 44234 | 0,34926 | 55345 | -0,48847262 |
| 21513 | 0,5924402 | 33124 | 0,5024883 | 44235 | 0,2283025 | 55351 | -0,2878794 |
| 21514 | 0,475908 | 33125 | 0,3815308 | 44241 | 0,4729671 | 55352 | -0,36306694 |
| 21515 | 0,3549505 | 33131 | 0,7024982 | 44242 | 0,3977796 | 55353 | -0,3826461 |
| 21521 | 0,6417299 | 33132 | 0,6273107 | 44243 | 0,3782004 | 55354 | -0,49917831 |
| 21522 | 0,5665423 | 33133 | 0,6077315 | 44244 | 0,2616682 | 55355 | -0,6201358 |
| 21523 | 0,5469632 | 33134 | 0,4911993 | 44245 | 0,1407107 | 55411 | -0,06849045 |
| 21524 | 0,430431 | 33135 | 0,3702418 | 44251 | 0,3413039 | 55412 | -0,14367799 |
| 21525 | 0,3094735 | 33141 | 0,6149064 | 44252 | 0,2661164 | 55413 | -0,16325715 |
| 21531 | 0,6304409 | 33142 | 0,5397188 | 44253 | 0,2465372 | 55414 | -0,27978936 |
| 21532 | 0,5552533 | 33143 | 0,5201397 | 44254 | 0,130005 | 55415 | -0,40074685 |
| 21533 | 0,5356742 | 33144 | 0,4036075 | 44255 | 0,0090475 | 55421 | -0,11396745 |
| 21534 | 0,419142 | 33145 | 0,28265 | 44311 | 0,601862 | 55422 | -0,18915499 |
| 21535 | 0,2981845 | 33151 | 0,4832432 | 44312 | 0,5266744 | 55423 | -0,20873415 |
| 21541 | 0,5428491 | 33152 | 0,4080557 | 44313 | 0,5070953 | 55424 | -0,32526636 |
| 21542 | 0,4676615 | 33153 | 0,3884765 | 44314 | 0,3905631 | 55425 | -0,44622385 |
| 21543 | 0,4480824 | 33154 | 0,2719443 | 44315 | 0,2696056 | 55431 | -0,12525645 |
| 21544 | 0,3315502 | 33155 | 0,1509868 | 44321 | 0,556385 | 55432 | -0,20044399 |
| 21545 | 0,2105927 | 33211 | 0,6965586 | 44322 | 0,4811974 | 55433 | -0,22002315 |
| 21551 | 0,4111859 | 33212 | 0,6213711 | 44323 | 0,4616183 | 55434 | -0,33655536 |
| 21552 | 0,3359983 | 33213 | 0,6017919 | 44324 | 0,3450861 | 55435 | -0,45751285 |
| 21553 | 0,3164192 | 33214 | 0,4852597 | 44325 | 0,2241286 | 55441 | -0,21284827 |
| 21554 | 0,199887 | 33215 | 0,3643022 | 44331 | 0,545096 | 55442 | -0,28803581 |
| 21555 | 0,0789295 | 33221 | 0,6510816 | 44332 | 0,4699084 | 55443 | -0,30761497 |
| 22111 | 0,8003066 | 33222 | 0,5758941 | 44333 | 0,4503293 | 55444 | -0,42414718 |
| 22112 | 0,7251191 | 33223 | 0,5563149 | 44334 | 0,3337971 | 55445 | -0,54510467 |
| 22113 | 0,7055399 | 33224 | 0,4397827 | 44335 | 0,2128396 | 55451 | -0,34451145 |
| 22114 | 0,5890077 | 33225 | 0,3188252 | 44341 | 0,4575042 | 55452 | -0,41969899 |
| 22115 | 0,4680502 | 33231 | 0,6397926 | 44342 | 0,3823166 | 55453 | -0,43927815 |
| 22121 | 0,7548296 | 33232 | 0,5646051 | 44343 | 0,3627375 | 55454 | -0,55581036 |
| 22122 | 0,6796421 | 33233 | 0,5450259 | 44344 | 0,2462053 | 55455 | -0,67676785 |
| 22123 | 0,6600629 | 33234 | 0,4284937 | 44345 | 0,1252478 | 55511 | -0,1842903 |
| 22124 | 0,5435307 | 33235 | 0,3075362 | 44351 | 0,325841 | 55512 | -0,25947784 |
| 22125 | 0,4225732 | 33241 | 0,5522008 | 44352 | 0,2506534 | 55513 | -0,279057 |
| 22131 | 0,7435406 | 33242 | 0,4770133 | 44353 | 0,2310743 | 55514 | -0,39558921 |
| 22132 | 0,6683531 | 33243 | 0,4574341 | 44354 | 0,1145421 | 55515 | -0,5165467 |
| 22133 | 0,6487739 | 33244 | 0,3409019 | 44355 | -0,0064154 | 55521 | -0,2297673 |
| 22134 | 0,5322417 | 33245 | 0,2199444 | 44411 | 0,5452299 | 55522 | -0,30495484 |
| 22135 | 0,4112842 | 33251 | 0,4205376 | 44412 | 0,4700424 | 55523 | -0,324534 |
| 22141 | 0,6559488 | 33252 | 0,3453501 | 44413 | 0,4504632 | 55524 | -0,44106622 |
| 22142 | 0,5807613 | 33253 | 0,3257709 | 44414 | 0,333931 | 55525 | -0,5620237 |
| 22143 | 0,5611821 | 33254 | 0,2092387 | 44415 | 0,2129735 | 55531 | -0,2410563 |
| 22144 | 0,4446499 | 33255 | 0,0882812 | 44421 | 0,4997529 | 55532 | -0,31624384 |
| 22145 | 0,3236924 | 33311 | 0,6810957 | 44422 | 0,4245654 | 55533 | -0,335823 |
| 22151 | 0,5242856 | 33312 | 0,6059082 | 44423 | 0,4049862 | 55534 | -0,45235521 |
| 22152 | 0,4490981 | 33313 | 0,586329 | 44424 | 0,288454 | 55535 | -0,5733127 |
| 22153 | 0,4295189 | 33314 | 0,4697968 | 44425 | 0,1674965 | 55541 | -0,32864812 |
| 22154 | 0,3129867 | 33315 | 0,3488393 | 44431 | 0,4884639 | 55542 | -0,40383566 |
| 22155 | 0,1920292 | 33321 | 0,6356187 | 44432 | 0,4132764 | 55543 | -0,42341482 |
| 22211 | 0,7376011 | 33322 | 0,5604312 | 44433 | 0,3936972 | 55544 | -0,53994703 |
| 22212 | 0,6624135 | 33323 | 0,540852 | 44434 | 0,277165 | 55545 | -0,66090452 |
| 22213 | 0,6428344 | 33324 | 0,4243198 | 44435 | 0,1562075 | 55551 | -0,4603113 |
| 22214 | 0,5263022 | 33325 | 0,3033623 | 44441 | 0,4008721 | 55552 | -0,53549884 |
| 22215 | 0,4053447 | 33331 | 0,6243297 | 44442 | 0,3256846 | 55553 | -0,555078 |
| 22221 | 0,6921241 | 33332 | 0,5491422 | 44443 | 0,3061054 | 55554 | -0,67161021 |
|  |  |  |  |  |  | 55555 | -0,7925677 |
