# Supplementary material for: Valuing health-related quality of life using a hybrid approach: Tunisian value set for the EQ-5D-3L
Source: Qual Life Res. 2021 Jan 14;30(5):1445–55. doi: 10.1007/s11136-020-02730-z (PMC8068700; doi:10.1007/s11136-020-02730-z)
Supplement: Supplementary file 4 — (DOCX 14 kb) [file 11136_2020_2730_MOESM4_ESM.docx]

For the random intercept models:

$$U_{ij}=\beta_{0}+\beta_{1}{MO2}_{j}+\beta_{2}{MO3}_{j}+\beta_{3}{SC2}_{j}+\beta_{4}{SC3}_{j}+\beta_{5}{UA2}_{j}+\beta_{6}{UA3}_{j}+\beta_{7}{PD2}_{j}+\beta_{8}{PD3}_{j}+\beta_{9}{AD2}_{j}+\beta_{10}{AD3}_{j}+\varepsilon_{ij}+\mu_{i}$$

Where $\mu_{j} \sim N(0,\sigma_{\mu}^{2})$. $i$ is the respondent, $j$ is the response, as each respondent completes 10 cTTO tasks. $\varepsilon_{ij} \sim N(0,\sigma_{j}^{2})$

For the logit model and the hybrid models:

$$U_{j}=\beta_{0}+\beta_{1}{MO2}_{j}+\beta_{2}{MO3}_{j}+\beta_{3}{SC2}_{j}+\beta_{4}{SC3}_{j}+\beta_{5}{UA2}_{j}+\beta_{6}{UA3}_{j}+\beta_{7}{PD2}_{j}+\beta_{8}{PD3}_{j}+\beta_{9}{AD2}_{j}+\beta_{10}{AD3}_{j}+\varepsilon_{ij}$$

For the Tobit model, we have the following extra equation:

$$U=\left\{ \begin{aligned} U" if U">-1 \\ -1 if U"<-1 \end{aligned} \right.$$

Where $U"$ is a latent variable that the tobit model assumes to be underlying of $U$, and the tobit model adjusts the parameter estimates based on this.

For all models that correct for heteroscedasticity (i.e. models 3,4, III and IV), we defined the following extra equation for the variance of the error term:

$$\sigma_{j}=exp(\gamma_{0}+\gamma_{1}{MO2}_{j}+\gamma_{2}{MO3}_{j}+\gamma_{3}{SC2}_{j}+\gamma_{4}{SC3}_{j}+\gamma_{5}{UA2}_{j}+\gamma_{6}{UA3}_{j}+\gamma_{7}{PD2}_{j}+\gamma_{8}{PD3}_{j}+\gamma_{9}{AD2}_{j}+\gamma_{10}{AD3}_{j})$$
